# Assessing an effective feeding strategy to optimize crude glycerol utilization as sustainable carbon source for lipid accumulation in oleaginous yeasts

**DOI:** 10.1186/s12934-016-0467-x

**Published:** 2016-05-05

**Authors:** Lorenzo Signori, Diletta Ami, Riccardo Posteri, Andrea Giuzzi, Paolo Mereghetti, Danilo Porro, Paola Branduardi

**Affiliations:** Department of Biotechnology and Biosciences, University of Milano-Bicocca, Piazza della Scienza 2, 20126 Milan, Italy; Department of Physics, University of Milano-Bicocca, Piazza della Scienza 3, 20126 Milan, Italy; Consorzio Nazionale Interuniversitario per le Scienze fisiche della Materia (CNISM), UdR Milano-Bicocca, Via R. Cozzi 53, 20126 Milan, Italy; Center for Nanotechnology Innovation@NEST, Istituto Italiano di Tecnologia, Piazza San Silvestro 12, 56127 Pisa, Italy

**Keywords:** *Cryptococcus curvatus*, *Rhodosporidium toruloides*, *Lipomyces starkeyi*, Crude glycerol, Fatty acids methyl esters (FAME), Flow-cytometry, Fourier transform infrared (FTIR) microspectroscopy, Principal component analysis (PCA)

## Abstract

**Background:**

Microbial lipids can represent a valuable alternative feedstock for biodiesel production in the context of a viable bio-based economy. This production can be driven by cultivating some oleaginous microorganisms on crude-glycerol, a 10 % (w/w) by-product produced during the transesterification process from oils into biodiesel. Despite attractive, the perspective is still economically unsustainable, mainly because impurities in crude glycerol can negatively affect microbial performances. In this view, the selection of the best cell factory, together with the development of a robust and effective production process are primary requirements.

**Results:**

The present work compared crude versus pure glycerol as carbon sources for lipid production by three different oleaginous yeasts: *Rhodosporidium toruloides* (DSM 4444), *Lipomyces starkeyi* (DSM 70295) and *Cryptococcus curvatus* (DSM 70022). An efficient yet simple feeding strategy for avoiding the lag phase caused by growth on crude glycerol was developed, leading to high biomass and lipid production for all the tested yeasts. Flow-cytometry and fourier transform infrared (FTIR) microspectroscopy, supported by principal component analysis (PCA), were used as non-invasive and quick techniques to monitor, compare and analyze the lipid production over time. Gas chromatography (GC) analysis completed the quali-quantitative description. Under these operative conditions, the highest lipid content (up to 60.9 % wt/wt) was measured in *R. toruloides*, while *L. starkeyi* showed the fastest glycerol consumption rate (1.05 g L^−1^ h^−1^). Being productivity the most industrially relevant feature to be pursued, under the presented optimized conditions *R. toruloides* showed the best lipid productivity (0.13 and 0.15 g L^−1^ h^−1^ on pure and crude glycerol, respectively).

**Conclusions:**

Here we demonstrated that the development of an efficient feeding strategy is sufficient in preventing the inhibitory effect of crude glycerol, and robust enough to ensure high lipid accumulation by three different oleaginous yeasts. Single cell and in situ analyses allowed depicting and comparing the transition between growth and lipid accumulation occurring differently for the three different yeasts. These data provide novel information that can be exploited for screening the best cell factory, moving towards a sustainable microbial biodiesel production.

**Electronic supplementary material:**

The online version of this article (doi:10.1186/s12934-016-0467-x) contains supplementary material, which is available to authorized users.

## Background

The progressive depletion of natural oil reserves, together with associated environmental concerns about greenhouse gas (GHG) emissions, has stimulated the development of sustainable process for the production of materials, chemicals and fuels from renewable resources.

Biodiesel is a renewable, safe and non-toxic energy source and a possible substitute of petroleum-based diesel [1, 2]. It is mainly produced through the *trans*-esterification of renewable feedstock, such as vegetable oils and animal fats, into fatty acid methyl esters (FAMEs).

However, to become an economically feasible alternative, biodiesel must compete in the market with petro-diesel fuel, but the actual high costs of production represent an obstacle for its commercialization. About 70–90 % of the overall costs depends on the feedstock price [3, 4]; furthermore, traditional oil-rich crops are limited by land availability, influenced by the climate and are in constant debate due to the food-versus-fuel issue [5].

An emerging potential alternative for biodiesel production is represented by microbial lipids, also referred as single-cell oils (SCOs; [6]), which could lead to a green and sustainable biodiesel production process, with no competition with the food supply chain.

Different oleaginous microorganisms have the ability to accumulate triacylglycerols (TAGs) as storage metabolites [7], with a fatty acid composition similar to that of many plant seed oils in terms of chain length and degree of unsaturation [8].

Among oleaginous microorganisms, yeasts exhibit advantages over bacteria, molds and algae, due to their higher growth rate, biomass and lipid productivities [9, 10]. Moreover, in comparison to plants and open-pond grown algae, yeasts can be easily grown in bioreactors (therefore not affected by season nor by climate), and the process has an easier scale-up [11].

In oleaginous microorganisms lipid accumulation is critically affected by the carbon-to-nitrogen (C/N) ratio and typically occurs under nitrogen limited conditions and in the presence of high sugar content [1]. When nitrogen is limiting, triglycerides are stored within intracellular lipid bodies [12].

The main oleaginous yeast genera so far identified include species belonging to the genera *Yarrowia*, *Candida*, *Rhodotorula*, *Rhodosporidium*, *Cryptococcus*, *Trichosporon* and *Lipomyces* [11]. Some oleaginous yeasts have been reported to accumulate lipids up to 80 % of their total dry cell weight under appropriate conditions [7, 11, 13].

However, the production of biodiesel from microbial feedstock remains economically unsustainable if expensive and edible substrates are considered [14]. The implementation with renewable waste raw materials (e.g. whey, crude glycerol, lignocellulosic biomass), having zero or even negative costs, could make microbial lipid production economically feasible. Indeed crude glycerol is the main byproduct, about 10 % (w/w), of the conversion of oils into biodiesel. In other words, for every 3 mol of methyl esters produced, 1 mol of glycerol is obtained as a byproduct [15]. Considering the increasing demand for biodiesel, larger amounts of glycerol are expected of being accumulated as a byproduct [16]. Nowadays, in some countries, crude glycerol is treated as industrial wastewater or simply incinerated, making biodiesel a “grey” fuel rather a green fuel alternative [17].

Despite desirable, an efficient valorization of crude glycerol is difficult to achieve since it contains several impurities such as residual methanol, NaOH, carry-over fat/oil, some esters, and minor amounts of sulfur compounds, proteins, and minerals [17]. Refined glycerol could be a valuable product, but once more the purification process is too costly and energy-intensive [18].

Nevertheless, crude glycerol has been tested in many studies as a substrate for the production of SCOs or for other metabolic compounds (such as citric acid, acetic acid, polyols, etc.) by several eukaryotic microbial strains [19].

In this study, the oleaginous yeasts *Cryptococcus curvatus*, *Lipomyces starkeyi* and *Rhodosporidium toruloides* were chosen as three of the most promising cell factories for lipid production using crude glycerol as sole carbon source [5, 18, 20]. Furthermore, data concerning this topic in these strains are still scarce in literature [5, 18, 19, 21–24]. Here we demonstrate that the development of an efficient, yet simple, feeding strategy is sufficient to avoid the detrimental effects deriving from the impurities present in crude glycerol and to enhance the production of lipids. This fermentation strategy greatly increased cell density as well as the rate of lipid production.

The lipid-producing capability of the chosen yeasts was investigated through the application of different techniques. In particular, fluorescent microscopy, flow-cytometry and FTIR microspectroscopy analyses were performed. All these are relatively fast approaches that do not require lipid extraction and would be helpful in the initial screening phase as well as in the real time evaluation of the effective production over fermentation time [25]. To the best of our knowledge, this is the first time that a combination of non-invasive techniques are applied to monitor lipid accumulation from crude glycerol over fermentation time.

Finally, gas chromatography analysis (GC) was performed to preliminarily qualify the potential of the obtained microbial oil as biodiesel.

## Results and discussion

### Effect of crude glycerol on yeast growth

It has been reported that different oleaginous yeast strains present different metabolic responses depending on the origin of the crude glycerol employed as carbon source [1]. In this study, the yeasts have been primarily selected based on literature data [20, 25, 26] and on oleaginous yeast strains available in our laboratory, searching for: high biomass and lipid accumulation, fast glycerol consumption and suitability for metabolic engineering approaches. This led to the selection of the following strains: *R. toruloides* (DSM 4444), *C. curvatus* (DSM 70022), *L. starkeyi* (DSM 70295), *Rhodotorula glutinis* (DSM 10134) and *Yarrowia lipolytica* (DSM 3286).

Afterwards, a second selection was performed by assessing the consumption of pure glycerol in shake flask experiments under nitrogen-limited conditions (data not shown). Based on their performances, *R. toruloides*, *C. curvatus* and *L. starkeyi*, were selected to further evaluate their potential for producing lipid from crude glycerol.

Five different defined media containing different proportions of crude versus pure glycerol were prepared (from A to E; see Methods for details). For all formulations, the initial concentration of total glycerol was fixed at 100 g L^−1^. Strains were cultivated in batch mode at 25 °C, 220 rpm, pH 5.5 (buffered with citric acid) for about 200 h, monitoring biomass (optical density and cell dry weight, Additional file 1: Table S1) and lipid accumulation (flow-cytometry and fluorescence microscopy analyses, by Nile Red staining, Additional file 1: Table S2).

**Fig. 1:**
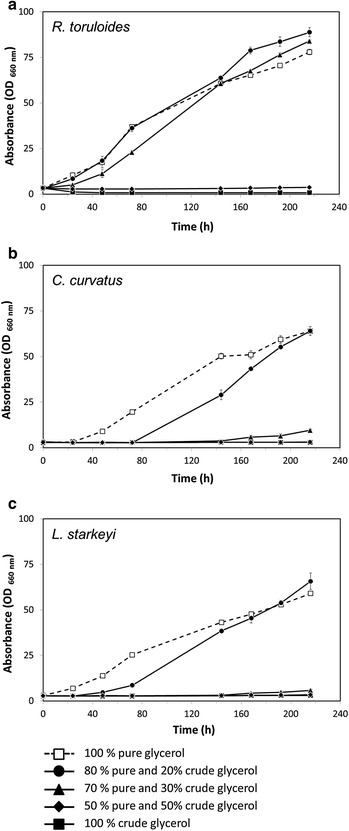
Growth profiles of *R. toruloides*, *C. curvatus* and *L. starkeyi* shake flasks cultivated with different concentrations of pure and crude glycerol. Growth curves (OD660 nm) of *R. toruloides* (**a**), *C. curvatus* (**b**) and *L. starkeyi* (**c**) cells at 25 °C and 220 rpm. Glycerol was used as sole carbon source at the final concentration of 100 g L^−1^. Five different mix of pure and crude glycerol were evaluated: 100 % pure glycerol (*A* *dashed line* and *unfilled square*), 80 % pure and 20 % crude glycerol (*B* *continuous line* and *filled circle*), 70 % pure and 30 % crude glycerol (*C* *continuous line* and *filled triangle*), 50 % pure and 50 % crude glycerol (*D* *continuous line* and *filled diamond*) and 100 % crude glycerol (*E* *continuous line* and *filled square*). Data are mean ± standard deviation (*error bars*) of three independent assays

As shown in Fig. 1, medium formulated with 100 g L^−1^ of pure glycerol (Medium A, dashed lines, open squares) well sustained the growth of all the strains selected for this study. On the contrary, no biomass increase was observed when crude glycerol concentrations of 50 (Medium D, closed diamonds) and 100 g L^−1^ (Medium E, closed squares) were used. On Medium C (30 g L^−1^ of crude glycerol; closed triangles) only *R. toruloides* showed a significant growth within 216 h. Instead, with the lowest crude glycerol concentration tested (20 g L^−1^; Medium B, closed circles), all the yeasts were able to reach, after 216 h, biomass values similar to those obtained on Medium A.

Between the tested yeasts, *R. toruloides* proved to be the most tolerant yeast (Fig. 1a). It also showed the highest biomass production, calculated as cell dry weight, within 216 h: about 30.3 g L^−1^ against only about 19.9 and 25.2 g L^−1^ reached with *C. curvatus* and *L. starkeyi*, respectively.

Overall, high crude glycerol concentrations inhibited yeast growth. The inhibition has mainly affected the duration of the lag phase, since the specific growth rates were similar or even higher of those observed on pure glycerol when crude glycerol was part of the medium composition (data not shown). An acidification of the medium as possible cause of the inhibition was excluded by monitoring the pH immediately after sampling. Indeed, batch bioreactor cultivations on 100 g L^−1^ of pure or crude glycerol confirmed the yeasts growth inhibition at the highest crude glycerol concentrations even after 240 h of cultivation, despite the fine control of the principal operative parameters (such as pH, air flow and dissolved oxygen; data not shown). These results confirmed that high glycerol concentrations are inhibitory also under controlled environment, where the conditions should promote microbial growth.

### Feeding pure vs crude glycerol for lipid production

Feeding strategies have been often proven to be crucial for the control of microbial cultures and production in biochemical processes [27]. Therefore, here we assessed the efficacy of developing a fed-batch protocol for minimizing the growth inhibition caused by the use of crude glycerol.

Briefly, strains were cultivated at 25 °C, pH 5.5 and monitored until glycerol exhaustion. The inlet gas flow rate was maintained constant at 1 vvm and stir was set in cascade to 25 % of dissolved oxygen to ensure fully aerobic conditions.

The cultivation was designed to have three different phases: (1) initial phase on pure glycerol (from 0 to 28 h) to adapt cells to the operative conditions; (2) “feeding” phase (from 28 to 48 h) where the medium was fed to reach a final concentration of about 100 g L^−1^ of glycerol (crude versus pure); (3) “lipid accumulation” phase under nitrogen limitation and in excess glycerol (from 48 h to the time of glycerol depletion) (see “Methods” section for details).

**Fig. 2:**
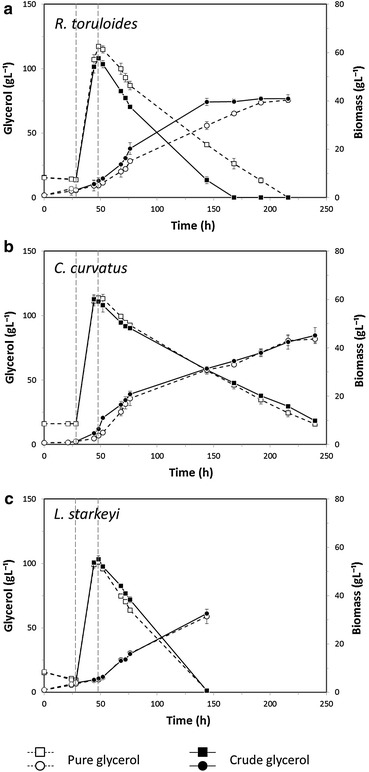
Glycerol consumption and growth (CDW) profiles of *R. toruloides*, *C. curvatus* and *L. starkeyi* under fed-batch cultivation. Growth (CDW; g L^−1^) and glycerol consumption profiles (g L^−1^) of *R. toruloides* (**a**), *C. curvatus* (**b**) and *L. starkeyi* (**c**) cultivated on pure (*dashed line*) and crude (*continuous line*) glycerol. Data are mean ± standard deviation (*error bars*) of three independent assays

Figure 2 (left panels) shows the glycerol consumption profiles of *R.toruloides* (a), *C. curvatus* (b) and *L. starkeyi* (c) on both pure (dashed line) and crude (continuous line) glycerol.

As reported in literature [28], the calculation of the optimal feeding rate is complex, since it has to match the requirements of the desired process of production. Preliminary experiments were performed testing different feeding rates (see Additional file 1: Figure S1 for details). From these experiments resulted that the highest feeding rate among the tested ones (0.57 mL min^−1^) led to a fast culture dilution and, consequently, to very low specific biomass production and specific glycerol consumption rates. Instead, a feeding rate of 0.32 mL min^−1^ resulted in a higher biomass accumulation, but at the expense of the lipid production and productivity, probably because of a slower transition from growth to the lipid accumulation phase.

Among the tested ones, the defined feeding rate of 0.45 mL min^−1^ (calculated as reported in Methods) was low enough to avoid an excessive culture dilution leading to a considerable lipid accumulation too. Nevertheless, it is important to notice that all the tested feeding rates allowed the crude glycerol consumption avoiding the growth inhibition previously described. From a general point of view, during the initial 24 h the glycerol utilization rate was low in all the three strains, associated with an adaptation to the operating conditions. With the start of the feeding (28 h) all the tested yeasts were able to grow on crude glycerol, demonstrating the efficacy and the robustness of the feeding strategy. The crucial role of the feeding strategy can be further appreciated considering the shake flask and bioreactor experiments previously described, where none of the selected yeasts proved to be able to grow starting with 100 g L^−1^ of crude glycerol.

In *C. curvatus* and *L. starkeyi* no significant differences were observed between the consumption profiles of pure and crude glycerol (Fig. 2b, c). Surprisingly, in *R. toruloides* crude glycerol consumption was markedly faster than that of pure glycerol (~0.73 and ~0.98 g L^−1^ h^−1^on pure and crude glycerol, respectively) (Fig. 2a).

Overall, the highest glycerol uptake rates were observed in *L. starkeyi* (~1.03 and ~1.05 g L^−1^ h^−1^ on pure and crude glycerol, respectively), while the lowest uptake rates were measured for *C. curtvatus* (~0.52 and ~0.48 g L^−1^ h^−1^ on pure and crude glycerol, respectively). This feature did not emerged during shake-flask batch experiments.

Table 1 reports a comparison of the cell mass and lipid productivities of the three yeasts growing on pure and crude glycerol. According to the data on substrates consumption, in *R. toruloides* also biomass production was markedly faster on crude glycerol compared to pure glycerol: at 144 h the dry cell weight on pure and crude glycerol was of 29.75 and 39.55 g L^−1^, respectively. These findings are in line with literature data reporting how crude glycerol can in some cases lead to higher biomass and lipid productivities when compared to pure substrate [18], due to the presence of macro elements, vitamins and other compounds that during the biodiesel production process can diffuse to the glycerol phase [29]. Possibly because of different nutritional requirements, biomass accumulation proceeded without significant differences between crude and pure glycerol cultures in *C. curvatus* and *L. starkeyi* (Table 1).

**Table 1:** Comparison of parameters related to biomass and lipid production among *R. toruloides*, *C. curvatus* and *L. starkeyi* grown on pure and crude glycerol

Yeast	Carbon source	Maximum DW (g L^−1^)	Biomass productivity (g L^−1^ h^−1^)^a^	Biomass yield (g g^−1^)	Glycerol uptake rate (g L^−1^ h^−1^)^a^	Lipid (%)	Lipid productivity (g L^−1^ h^−1^)
*R. toruloides*	Pure glycerol	40.4	0.21	0.33	0.73	60.9	0.13
	Crude glycerol	41.0	0.24	0.36	0.98	60.0	0.15
*C. curvatus*	Pure glycerol	43.7	0.21	0.38	0.52	46.9	0.10
	Crude glycerol	45.1	0.18	0.37	0.48	50.9	0.09
*L. starkeyi*	Pure glycerol	31.4	0.31	0.26	1.03	48.2	0.10
	Crude glycerol	32.7	0.29	0.26	1.05	55.9	0.13

Data shown are the mean of three independent experiments where the deviation from the mean value was less than 5 %

^a^Biomass productivity as well as glycerol uptake rate were calculated starting to the end of the feeding

The highest biomass productions were achieved in *C. curvatus* (~43.7 and ~45.1 g L^−1^ on pure and crude glycerol, respectively) but, independently from the substrate (pure or crude glycerol), associated with the lowest productivity compared to the other strains. These data underline once more that both production and productivity can vary considerably depending on the selected cell factory.

Regarding the production of interest, remarkably this process set-up assured in the three investigated yeasts a good lipid accumulation (Table 1). In particular, *R. toruloides* was found to be the most effective in terms of lipid productivity and overall lipid content (on dry cell weight basis): on crude glycerol a lipid productivity of 0.15 g L^−1^ h^−1^, very close to the one obtained with pure glycerol (0.13 g L^−1^ h^−1^), and almost the same lipid content (60.9 and 60.0 % on pure and crude glycerol, respectively) were obtained.

Table 2 presents an overview of literature data regarding total dry weight and microbial oil content achieved during cultivation of *R. toruloides, C. curvatus* and *L. starkeyi* yeast strains on glycerol.

**Table 2:** Literature-cited results of *R. toruloides*, *C. curvatus* and *L. starkeyi* strains cultivated on various crude glycerol-based media during growth under various fermentation configurations and their comparisons with the present study

Strain	Carbon source	Nitrogen source	Dry weight (g L^−1^)	Lipid (%)	Lipid productivity (g L^−1^ h^−1^)	Cultivation mode	Glycerol (g L^−1^)	Reference
*R. toruloides* Y4	Crude glycerol	Hydrolysate from rapeseed meal	31.1	41.7	0.11	Fed-batch; 1-L bioreactor	–	[22]
			19.3	43.0	0.07	Batch; 1-L bioreactor	100	
	Pure glycerol		35.3	46.0	0.14	Batch; 1-L bioreactor	100	
			43.0	45.8	0.17	Batch; 1-L bioreactor	100	
*R. toruloides* Y4	Crude glycerol	Peptone and yeast extract	20.3	42.5	0.07	Flask	100	[24]
	Pure glycerol		21.1	40.3	0.07	Flask	100	
*R. toruloides* AS2.1389	Crude glycerol	(NH_4_)_2_SO_4_ and yeast extract	19.2	47.7	0.06	Flask	50	[18]
			20.1	42.9	0.05	Flask	50	
			26.7	69.5	0.09^a^	Batch; 5-L bioreactor	60	
			18.00	74.1	0.07^a^	Batch; 5-L bioreactor	60	
*R. toruloides* NRRL Y-27012	Crude glycerol	Peptone and yeast extract	30.1	40.0	0.03	Flask	120	[19]
			23.8	47.0	0.04	Flask	95	
*R. toruloides* DSM 4444	Crude glycerol	SFM hydrolysate	47.9	37.8	0.14	Fed-batch; 3.6-L bioreactor	–	[23]
		PSFM hydrolysate	37.4	51.3	0.17	Fed-batch; 3.6-L bioreactor	–	
*C. curvatus* ATCC 20508	Crude glycerol	Corn steep liquor, baker’s yeast autolysate and malt extract	50.4	45.0	0.17	Fed-batch; 30-L bioreactor	–	[5]
			58.9	43.0	0.19	Fed-batch; 6-L bioreactor	–	
			52.3	43.0	0.16	Fed-batch; 6-L bioreactor	–	
			69.2	40.0	0.24	Fed-batch; 6-L bioreactor	–	
*C. curvatus* ATCC 20509	Crude glycerol	Yeast extract	32.9	52.9	0.06	Two-stage fed-batch; 2-L biorector	–	[21]
*C. curvatus* ATCC 20509	Crude glycerol	SFM hydrolysate	38.0	47.1	0.09	Fed-batch; 3.6-L bioreactor	–	[23]
		PSFM hydrolysate	34.6	50.0	0.11	Fed-batch; 3.6-L bioreactor	–	
*L. starkeyi* DSM 70296	Crude glycerol	Peptone and yeast extract	34.4	35.9	0.03	Flask	120	[19]
			23.3	35.0	0.02	Flask	100	
*R. toruloides* DSM 4444	Pure glycerol	(NH_4_)_2_SO_4_ and yeast extract	40.4	60.9	0.13	Batch with feeding; 2-L biorector	100	This study
	Crude glycerol		41.0	60.0	0.15	Batch with feeding; 2-L biorector	100	
*C. curvatus* DSM 70022	Pure glycerol	(NH_4_)_2_SO_4_ and yeast extract	43.7	46.9	0.10	Batch with feeding; 2-L bioreactor	100	This study
	Crude glycerol		45.1	50.9	0.09	Batch with feeding; 2-L biorector	100	
*L. starkeyi* DSM 70295	Pure glycerol	(NH_4_)_2_SO_4_ and yeast extract	31.4	48.2	0.10	Batch with feeding; 2-L biorector	100	This study
	Crude glycerol		32.7	55.9	0.13	Batch with feeding; 2-L biorector	100	

^a^Calculated considering fermentation time 195 h

In the case of *R. toruloides* strains, the CDW and lipid concentrations achieved in this study are among the highest reported in literature. To the best of our knowledge, on crude glycerol only in one case [23] a higher lipid productivity has been so far observed.

For *C. curvatus*, even if lipid content (%) is in line with those of other reports, lipid productivity is lower than what reported in [5].

Finally, for *L. starkeyi* a scarce number of reports has indicated lipid accumulation based on crude glycerol consumption. Among these, the highest CDW and lipid concentrations as well as productivity have been achieved in this study (Table 2).

Nevertheless and for sake of clarity, it is important to notice that a direct comparison of the literature data is difficult: as shown in Table 2, various fermentation configurations, as well as nitrogen source and glycerol concentrations were tested.

### Evaluation of lipid production in oleaginous yeasts by Nile red staining

Nile Red (NR) is a red phenoxazine dye, present as a minor component of commercial preparations of the non-fluorescent stain Nile Blue, which selectively stains lipophilic substances.

**Fig. 3:**
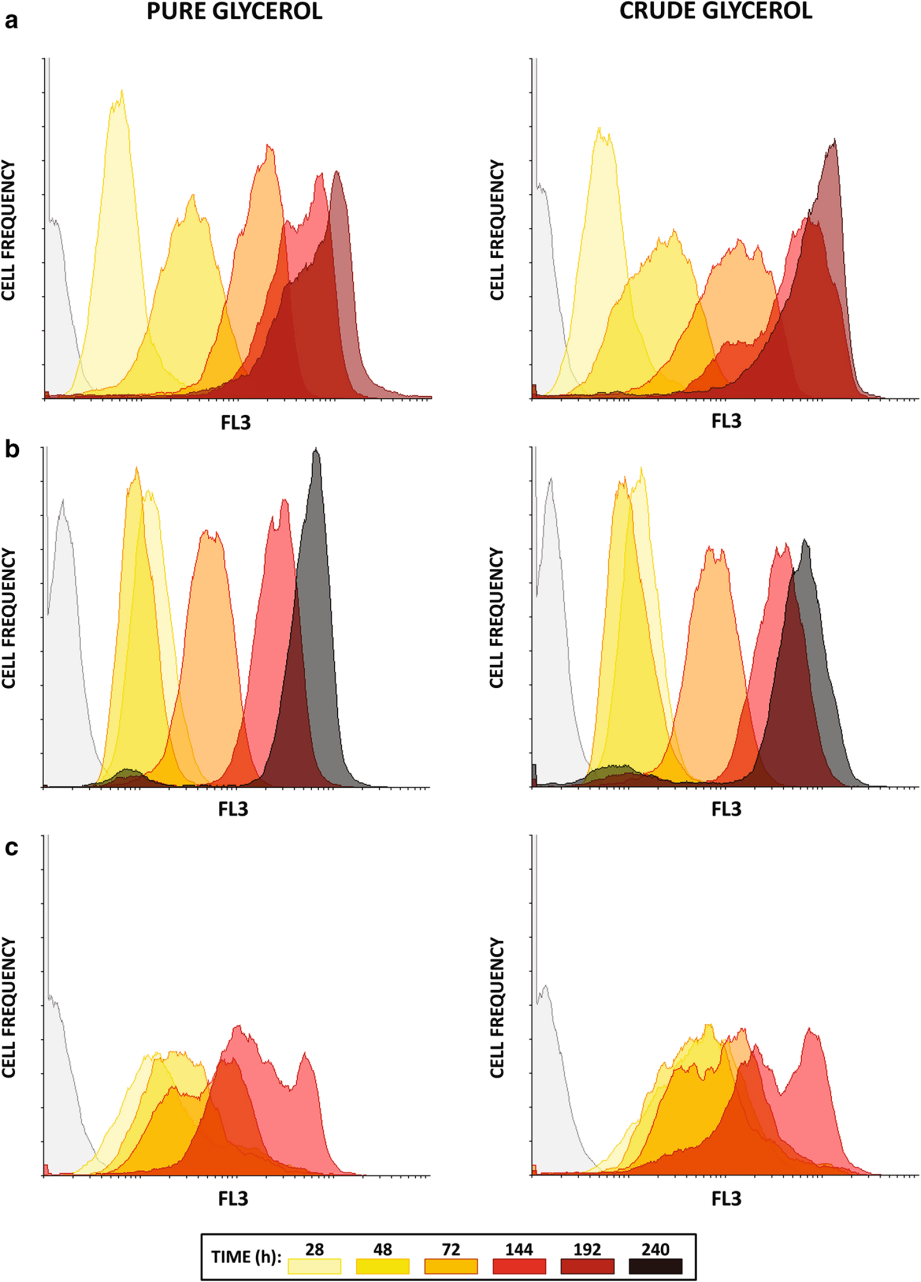
Flow-cytometry analysis of *R. toruloides*, *C. curvatus* and *L. starkeyi*. Overlaid histograms of *R. toruloides* (**a**), *C. curvatus* (**b**) and *L. starkeyi* (**c**) cells grown on pure (*left panels*) and crude glycerol (*right panels*) stained with Nile Red and analyzed through flow-cytometry after 28, 48, 72, 144, 192 (only *R. toruloides* and *C. curvatus*) and 240 h (only *C. curvatus*). The fluorescence emission was measured in the FL3 channel (>650 nm corresponding to polar lipids). For each condition, an example of control (cells not stained) is reported. Results shown are representative of three independent experiments, where the deviation from the X mean value was always less than 5 %

During fed-batch experiments cells were collected at different time points (T = 0, T = 28, T = 48, T = 72, T = 144, T = 192, T = 240 h after inoculation, see Fig. 3), stained with Nile Red and analyzed by flow-cytometry to evaluate the lipid content (see “Methods” section for details). In Fig. 3, the overlay histograms of *R. toruloides* (a), *C. curvatus* (b) and *L. starkeyi* (c) cells growing on both pure (left panels) and crude (right panels) glycerol are reported.

For all the tested strains, once started, lipid accumulation proceeded along with the fermentation as shown by an increase of fluorescence over time, with only slight variations from pure and crude glycerol cultures (Fig. 3). This increase was negligible in the non-oleaginous yeast, *S. cerevisiae*, (data not shown). It is interesting to note that, at time points near to glycerol exhaustion, Nile Red fluorescence signals related to crude glycerol cultivations were higher compared to those of pure glycerol cultures.

Comparing the different yeasts, fluorescence signals were low at 28 h for *R. toruloides* and *C. curvatus* (Fig. 3a, b), as expected. On the contrary, in *L. starkeyi* at 28 h fluorescence signals were higher (Fig. 3c), suggesting that lipid accumulation was already started during the shake-flask cultivation (pre-inoculum phase). The Nile Red fluorescence signals collected immediately after the inoculum (T = 0 h) were not showed since they were found to be very similar to those collected at 28 h. After 72 h from the inoculum the higher increase in the Nile Red fluorescence signals was observed in *R. toruloides* (Fig. 3): the FL3 X mean values increased from 9.0 and 9.3 (for pure and crude glycerol, respectively) at 28 h to 174.9 and 153.1 at 72 h (for pure and crude glycerol, respectively). Interestingly, a bimodal distribution was observed at 72 and 144 h in *L. starkeyi*, suggesting the existence of two distinct populations with different lipid content.

Overall, it can be noticed that, independently from the cultivation conditions (pure or crude glycerol), the higher fluorescence signals were observed in *R. toruloides* (Fig. 3a).

**Fig. 4:**
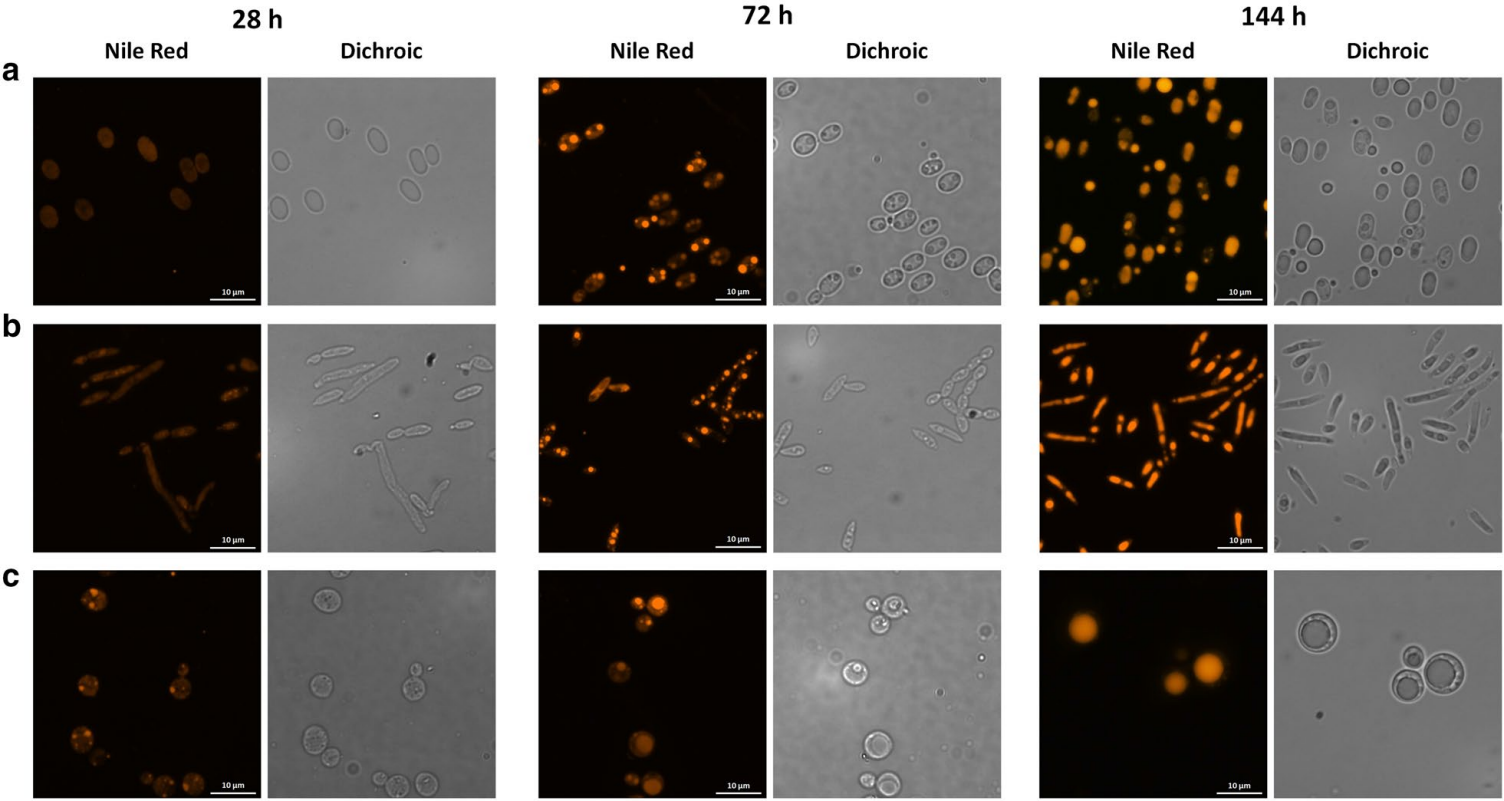
Fluorescence microscope analysis of *R. toruloides*, *C. curvatus* and *L. starkeyi*. *R. toruloides* (**a**), *C. curvatus* (**b**) and *L. starkeyi* (**c**) cells were stained with Nile Red and observed under the microscope after 28, 72, 144 h. For each condition, fluorescence images and the corresponding dichroic image is reported. Since no significant differences in lipid bodies shape and number were observed between samples derived from pure and crude glycerol cultivations, the images here reported refers only to the first condition. *Bar* indicates 10 μm

Fluorescence microscopy images confirmed flow-cytometry observations: as example, at 28 h *R. toruloides* and *C. curvatus* showed very weak fluorescence signals, while small lipid droplets were already visible in *L. starkeyi* cells (Fig. 4).

The direct observation of cells also enabled the detection of differences in lipid bodies size, morphology and abundance among the different yeasts, as previously reported but for different growth conditions [30].

*Rhodosporidium toruloides* (Fig. 4a) cells, with small lipid bodies at 72 h, modified into bigger cells (up to 5 μm) with one or two lipid bodies at 144 h. *C. curvatus* (Fig. 4b) at 72 h showed numerous small lipid bodies (less than 1 μm diameter) contained in long rod-shaped cells. After 144 h of cultivation, the majority of the cells contained only one or two bigger lipid bodies (3–4 μm) and some smaller ones (less than 1 μm). *L. starkeyi* (Fig. 4c) produced one or two large lipid bodies in their spherical cells (as shown in [31]). The small lipid bodies (less than 1 μm diameter), which were already visible in the cells at the beginning of the feeding phase (28 h), increased in size to more than 6 μm diameter after 144 h of cultivation.

Overall, in both dichroic and fluorescence microscopy images small lipid droplets, when present, were easily observed at early time points and they seemed to collapse into bigger structures over time.

Fluorescent microscopy analyses did not shed light on the formation in *L. starkeyi* of two sub-populations with different fluorescent signal, but overall they clearly depict the different transition between growth and lipid accumulation occurring among the three yeasts and provide a useful semi-quantitative evaluation of their lipid accumulation over time.

### FTIR microspectroscopy coupled to PCA for monitoring lipid accumulation in intact oleaginous yeast cells

Fourier Transform Infrared (FTIR) microspectroscopy has been demonstrated as a powerful, non-invasive and time-saving technique to monitor lipid accumulation in intact cells [25]. Indeed, this label-free spectroscopic approach allows to obtain a biochemical fingerprint of the sample under investigation, giving information on its main biomolecule content (see Additional file 1: Figure S2) [32].

**Fig. 5:**
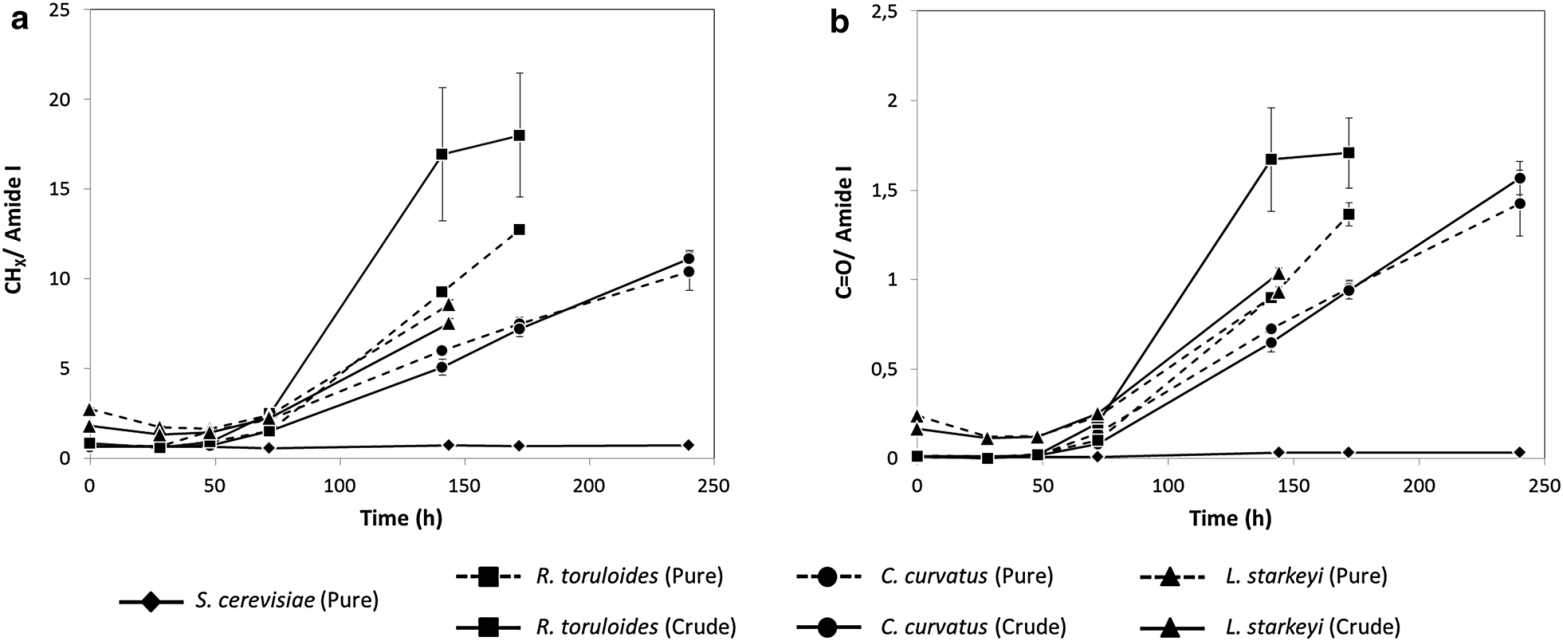
Time dependence of fatty acid production by Fourier transform infrared (FTIR) microspectroscopy analysis. Time dependence of the CHx stretching band area (**a**) and of the ester C = O (**b**) of *S. cerevisiae* (*filled diamond*)*, R. toruloides* (*filled square*), *C. curvatus* (*filled circle*) and *L. starkeyi* (*filled triangle*) cells growing on pure (*dashed lines*) and crude glycerol (*continuous lines*). Values were normalized for the total protein content given by the amide I band area

In this study we measured the infrared absorption of *R. toruloides*, *C. curvatus*, *L. starkeyi* and *S. cerevisiae* (as internal control) intact cells, at different times of growth (T = 0, T = 28, T = 48, T = 72, T = 144, T = 192, T = 240 h after inoculation). To evaluate the time course of lipid accumulation, in Fig. 5a is reported the temporal evolution of the CH_x_ stretching band area, between 3050 and 2800 cm^−1^ [33, 34], after normalization for the total protein content given by the amide I band area (see Additional file 1: Figure S2) [35]. We have found that lipid accumulation started approximately at 48 h of growth, with the exception of *L. starkeyi* (triangles) that, as detected also by Nile Red fluorescence (see Figs. 3c, 4c), already started to accumulate in the pre-inoculum phase. Also FTIR analysis confirmed the highest lipid accumulation in *R. toruloides* (squares), under crude glycerol feeding. These results have been also confirmed by the temporal evolution of the ester carbonyl band area, between 1760 and 1730 cm^−1^ [33, 34], again normalized for the total protein content (see Fig. 5b), in agreement with Nile Red staining and analysis.

**Fig. 6:**
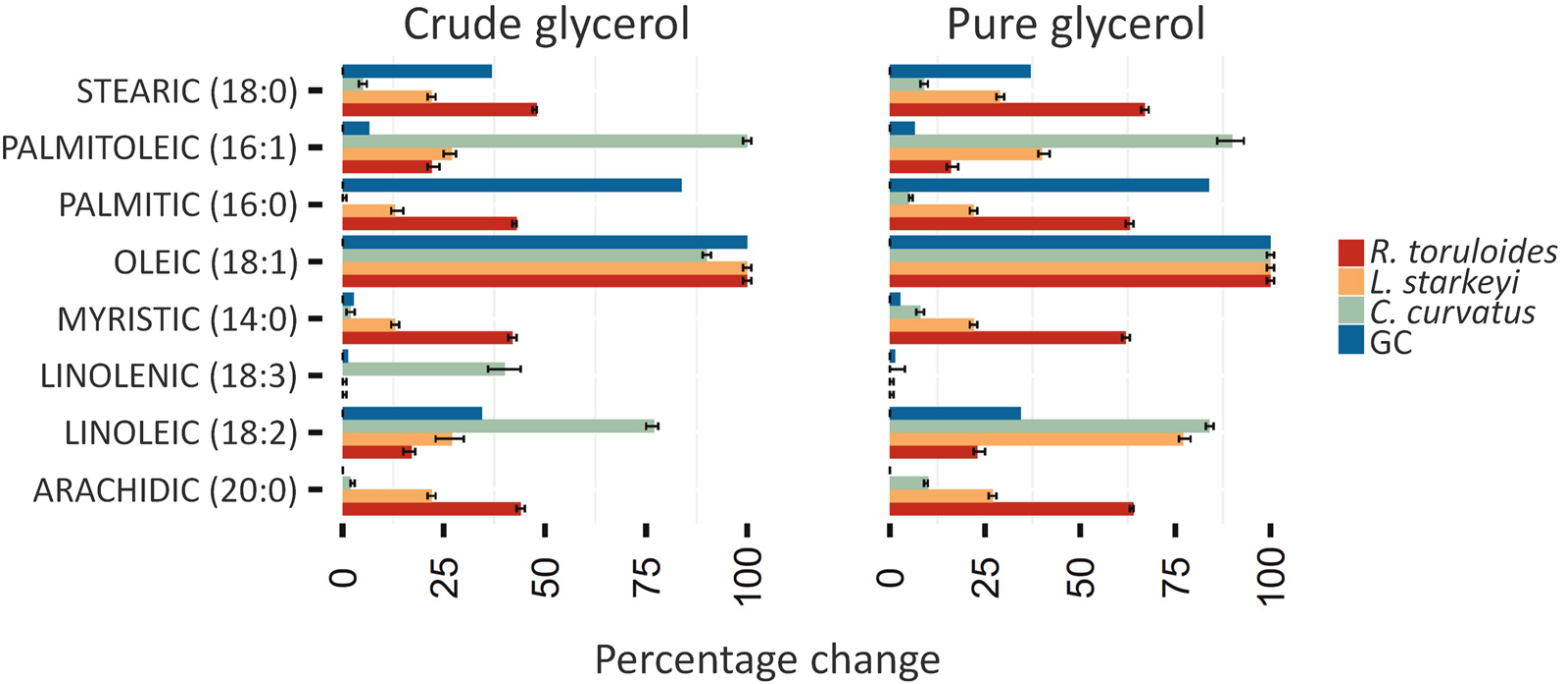
Average percentage changes of the selected fatty acids in intact cells. PCA results, obtained by the analysis of the FTIR spectra of intact cells, are summarized as percentage changes averaged across the three ranges ($\tilde{\Updelta }\left( {K_{144h;0h} ,S} \right),$ see “Methods” section for details), for *R. toruloides*, *L. starkeyi*, *C. curvatus* grown on pure glycerol (*left panel*) and crude glycerol (*right panel*). The larger the value, the more a lipid standard contributes to the spectral profile changes of the sample at 144 h compared to the time 0. In addition, the average (across range) value of the gas chromatographic (GC) data is shown scaled in the range 0–100 for comparison. *Error bars* indicate the bootstrapped 95 % confidence intervals

To better investigate the spectral features due to lipid accumulation and in particular to assign them to specific lipid molecules, we supported the infrared characterization with a multivariate analysis approach, namely the principal component analysis (PCA) [36]. In particular, we compared the IR response of the studied yeast strains with that of standard fatty acids, chosen among the most representative products of the oleaginous yeasts [1]. We performed the PCA on the raw spectra of samples taken at time 0 and 144 h, as representative of the yeast time-dependent behavior plus the raw spectra of the standard lipids. The spectral range was split into three ranges and the PCA was performed independently on each range. The Euclidean distance on the PCA score plot (Additional file 1: Figures S4, S5, S6) among the lipids standards and a given oleaginous yeast was used as a measure to quantify the accumulation of a class of lipids in a specific yeast. The distance was calculated for both time 0 and time 144 h, and a percentage change was then computed (see Additional file 1: Figure S3 for details). The procedure was repeated for each yeast strain grown in pure glycerol as well as in crude glycerol and for each analyzed spectral range. Finally, an average percentage change across the ranges was computed and shown in Fig. 6 (see “Methods” section and Additional file 1 for details about the procedure).

### FTIR analysis of the cell wall modifications during lipid accumulation

Lipid extraction from cells is one of the main drawbacks of biodiesel production since it is generally detrimental in terms of yields and costs. In particular, this phase is particularly tedious as oleaginous yeast cell wall becomes more and more difficult to break while cells are accumulating fatty acids. In this context, the analysis of the second derivative spectra allowed to characterize the IR response of the yeast cell wall carbohydrates (see Additional file 1: Figure S2) and in particular to investigate their modifications during the accumulation of lipids.

To this aim, we analyzed the spectral range between 1200–950 cm^−1^, due to the overlapping absorption of C–O vibrations mainly from carbohydrates, of O–P–O groups, and of C–O–P modes typical of phosphate esters [37, 38].

**Fig. 7:**
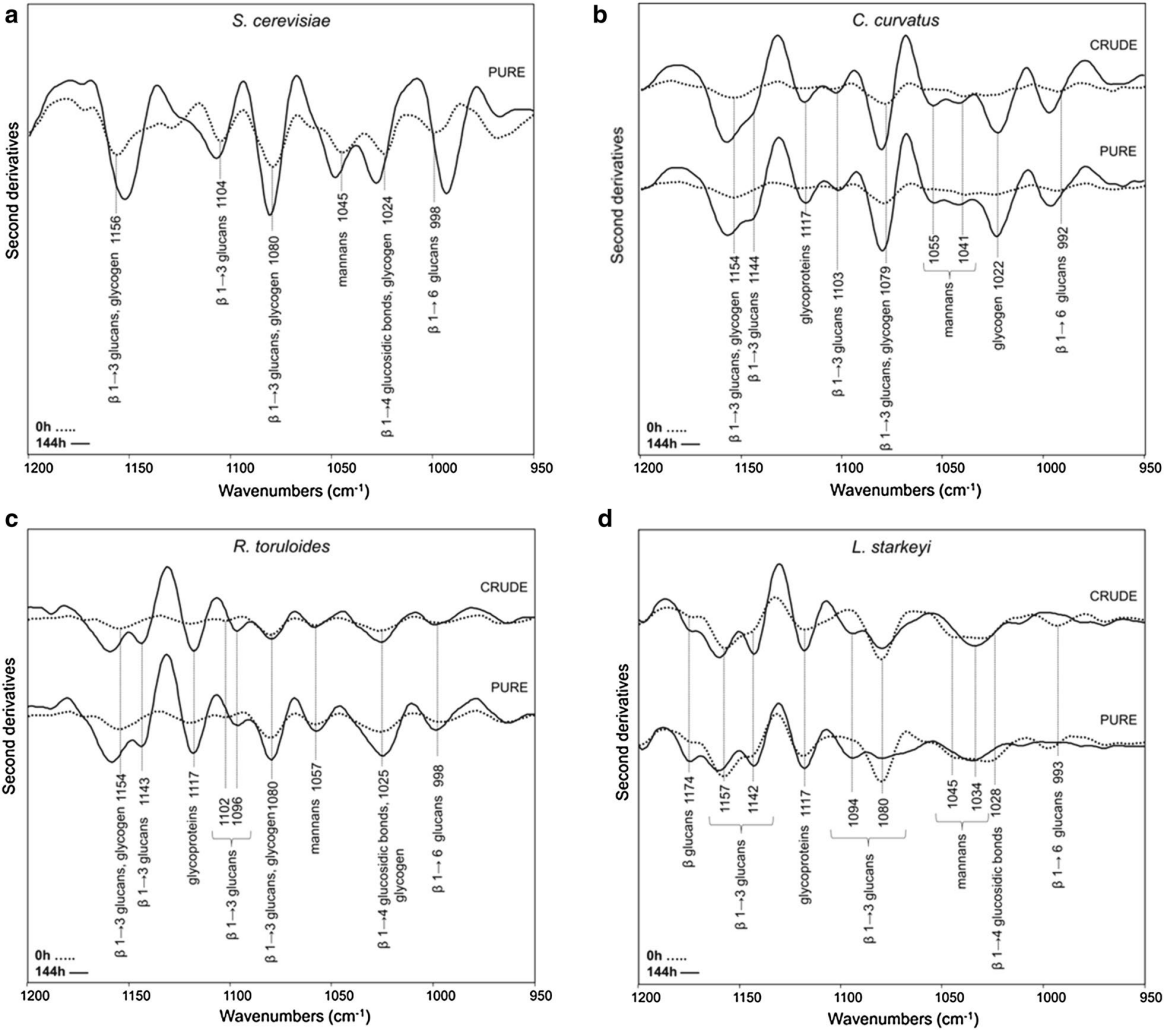
FTIR analysis of yeast cell wall modifications during lipid accumulation. The second derivatives of the FTIR spectra of yeast cells are reported in the spectral range comprised between 1200–950 cm^−1^, mainly ascribable to the absorption of cell wall carbohydrates. Spectra of control (**a**) and oleaginous yeasts *C. curvatus* (**b**) *R. toruloides* (**c**), and *L. starkeyi* (**d**) are displayed at time 0 and at 144 h of growth in crude and pure glycerol. For comparison, the second derivative spectra have been normalized at the tyrosine band at ~1516 cm^−1^

Firstly, in Fig. 7a the second derivative spectra of the control strain (*S. cerevisiae)* at

0 and 144 h are reported. At time 0, the spectrum is dominated by the absorption of glucans and mannans, major components of the yeast cell wall [39]. In particular, the absorptions at ~1156, 1104 and 1080 cm^−1^ can be mainly assigned to β 1 → 3 glucans, while the band at ~1024 cm^−1^ to β 1 → 4 glucosidic bonds in chitin and/or in glucans linked to a N-acetylglucosamine molecule; moreover, the component at ~998 cm^−1^ can be mostly due to β 1 → 6 glucans [37, 38, 40]. It should be noted that the simultaneous presence of the three bands at 1156, 1080 and 1024 cm^−1^ could be also indicative of the presence of glycogen [40]. Finally, the absorption at ~1045 cm^−1^ is mainly due to mannans [37, 38]. These components were found to increase in intensity after 144 h of growth, as displayed. Moreover, some bands were found to shift in peak position, including the ~1156 cm^−1^ that downshifted to ~1152 cm^−1^, the ~1024 cm^−1^ that upshifted to ~1028 cm^−1^, and the ~998 cm^−1^ that downshifted to ~993 cm^−1^. Overall, these variations could reflect a modification of carbohydrate interactions with the surrounding molecules of the cell wall.

Analogously, we investigated possible variations of the cell wall features during the accumulation of lipids in the oleaginous yeasts. At time 0 the spectrum of *C. curvatus* grown in medium supplemented with crude glycerol is mainly characterized by the β 1 → 3 glucan components at ~1154 and 1079 cm^−1^ (Fig. 7b). Moreover, a very low intensity band was detected around 1041 cm^−1^ that can be attributed to the absorption of mannans and a weak absorption at ~992 cm^−1^, mostly due to β1 → 6 glucans, was also observed. Interestingly, these spectral components were found to dramatically increase in intensity at 144 h, reflecting a profound rearrangement of the cell wall architecture during lipid accumulation. Furthermore, new spectral features were observed. In particular, beside the upshift of the β 1 → 3 glucans band up to ~1156 cm^−1^, a new component at ~1144 cm^−1^ was detected, again assigned to β 1 → 3 glucans. The appearance of new spectral features in the β 1 → 3 glucan absorption could be indicative of a modification of the physico-chemical properties of the complex network formed by glucans, which indeed interact with different cell wall components, including other carbohydrate polymers and/or proteins [39, 40]. In particular, modifications of glucan interactions with the surrounding wall components could lead to variations of their IR response. Moreover, a well resolved peak was observed at ~1117 cm^−1^, negligible at T = 0, that can be tentatively assigned to the O–H bending vibration of glycoproteins [41]. In addition, the component at ~1055 cm^−1^, due to mannans, was also detected. Interestingly, the glycogen absorption at ~1156, 1080, 1022 cm^−1^, negligible at T = 0, was found at high intensity at T = 144.

As also reported in the figure, very similar spectral features were observed when cells grew in medium supplemented with pure glycerol.

The analysis of cell wall carbohydrates in *R. toruloides* (Fig. 7c) at T = 0 in medium supplemented with crude as well as pure glycerol highlighted similar spectral features to those found for *C. curvatus* (Fig. 7b). During the lipid accumulation up to 144 h, in particular the β glucan bands and the glycogen absorption were found to increase in intensity and their increase was slightly higher in the case of cells grown in medium supplemented with pure glycerol. We should note that in *R. toruloides* the intensity variations of the β glucan bands were lower compared to those depicted in *C. curvatus* and also that the mannan absorption around 1041 cm^−1^, observed in *C. curvatus*, was not detected in the case of *R. toruloides*. Moreover, the broad band at ~1025 cm^−1^, that can be due to the overlapping absorption of glycogen and β 1 → 4 glucosidic bonds, was found to increase in intensity up to 144 h, particularly in pure medium.

Interestingly, the spectral features of *L. starkeyi* cell wall carbohydrates appeared to be different compared to the other analyzed oleaginous yeasts. In fact, at T = 0 the spectrum of *L. starkeyi*, grown in crude glycerol, is characterized firstly by a complex absorption band with three peaks at 1174, 1157, and 1142 cm^−1^ (Fig. 7d). While the latter two are due to β1 → 3 glucans, the 1174 cm^−1^ band can be tentatively assigned to β glucans [42]. Furthermore, the glycoprotein component at ~1117 cm^−1^ [41] is present in the T = 0 spectrum, together with the β1 → 3 glucan absorption at ~1080 cm^−1^. In addition, three low intensity bands were observed at ~1045 cm^−1^, due to mannans, and at ~ 1028 and ~ 993 cm^−1^, that can be respectively assigned to β1 → 4 glucosidic bonds and β1 → 6 glucans.

These spectral behaviors were found to change at 144 h, and in particular we observed a rearrangement of the β 1 → 3 glucan components. Indeed, a new component at ~1094 cm^−1^ appeared, accompanied by an intensity increase of the ~1142 cm^−1^ absorption and a reduction of the ~1080 cm^−1^ band. Moreover, a well resolved component at ~1034 cm^−1^, that can be assigned mainly to mannans [42] was observed, instead of the two bands at ~1045 and ~1028 cm^−1^ detected at T = 0. These results, which have been also found for the growth in medium added with pure glycerol, suggest that modifications of the cell wall physico-chemical properties occurred during lipid accumulation, involving the different carbohydrate components. However, in *L. starkeyi* we did not detected dramatic band intensity variations between 0 and 144 h, as instead seen for the other analyzed strains. This result could be due to the fact that cell wall modifications likely took place already in the pre-inoculum phase, when lipid accumulation has also started.

All this considered, the analysis of the cell wall modifications during the lipid accumulation indicates that the use of crude or pure glycerol as carbon source resulted in very similar structure and content of cell wall carbohydrates.

### Gas chromatography analysis of fatty acid composition

The lipids produced by fed-batch experiments with *R. toruloides*, *C. curvatus* and *L. starkeyi* at 144 h were hydrolyzed and transmethylated. The resulting fatty acid methyl esters (FAME) were then analyzed by gas chromatography (Table 3, see “Methods” section for details). Long-chain fatty acids were predominantly represented in all the strains and the four that accounted for over 90 % of the total were palmitic (C16:0), stearic (C18:0), oleic (C18:1) and linoleic acid (C18:2). In agreement with the FTIR-PCA results, the oleic acid was the most produced, while the linolenic acid was accumulated in very small quantities by all yeasts (see also Fig. 6). It should be noted, however, that for some fatty acids significant differences have been found between the results obtained with the FTIR-PCA and the GC approaches, likely due to the fact that intact cells and extracted lipids were analysed, respectively. Moreover, multivariate analysis could not allow to identify all the contributions of the different classes of fatty acids that are overlapped in the FTIR spectra of whole cells, instead disclosed by GC analyses.

**Table 3:** Gas chromatography analysis of *R. toruloides*, *C. curvatus* and *L. starkeyi* lipids produced using crude and pure glycerol as a sole carbon source

Yeast	Carbon source	Fatty acids composition (% wt/wt)										
		C 14:0	C 16:0	C 16:1	C 18:0	C 18:1	C 18:2	C 18:3	Other	S	M	P
*R. toruloides*	Pure glycerol	1.5 ± 0.1	27.9 ± 0.1	2.1 ± 0.1	12.3 ± 0.1	35.3 ± 0.1	17.4 ± 0.2	2.8 ± 0.1	0.7 ± 0.1	42.4 ± 0.2	37.4 ± 0.1	20.2 ± 0.2
	Crude glycerol	1.5 ± 0.1	27.5 ± 0.1	2.0 ± 0.3	12.5 ± 0.2	37.8 ± 0.1	15.8 ± 0.2	2.2 ± 0.1	0.7 ± 0.1	42.2 ± 0.1	39.8 ± 0.4	18.0 ± 0.3
*C. curvatus*	Pure glycerol	1.6 ± 0.3	29.6 ± 0.1	3.7 ± 0.3	18.6 ± 0.1	27.6 ± 0.2	14.9 ± 0.1	1.9 ± 0.2	2.1 ± 0.6	51.9 ± 0.1	31.3 ± 0.1	16.8 ± 0.2
	Crude glycerol	1.6 ± 0.3	30.6 ± 0.1	4.0 ± 0.3	15.4 ± 0.1	31.2 ± 0.1	14.5 ± 0.1	1.6 ± 0.1	1.1 ± 0.1	48.7 ± 0.4	35.2 ± 0.4	16.1 ± 0.1
*L. starkeyi*	Pure glycerol	3.1 ± 0.3	31.0 ± 0.1	4.3 ± 0.1	12.9 ± 0.1	39.4 ± 0.3	7.6 ± 0.1	0.5 ± 0.1	1.2 ± 0.3	48.2 ± 0.4	43.7 ± 0.3	8.1 ± 0.1
	Crude glycerol	3.0 ± 0.2	32.2 ± 0.1	4.2 ± 0.1	10.9 ± 0.1	40.9 ± 0.1	7.2 ± 0.1	0.6 ± 0.1	1.0 ± 0.2	47.1 ± 0.1	45.1 ± 0.1	7.8 ± 0.2

Peak areas less than 0.3 % were considered insignificant

Data are mean ± standard deviation of three independent assays

*S* saturated; *M* monounsaturated; *P* polyunsaturated

From a general point of view, the fatty acid profiles showed slight changes between the different culture conditions (pure *vs* crude glycerol). However, for all the tested yeasts, on crude glycerol the oleic acid content was slightly higher than that obtained from pure glycerol.

Table 3 details some interesting differences among the yeasts object of this study. In *R. toruloides* and *C. curvatus* at 144 h the percentage of 18:2 was about twice the percentage observed in *L. starkeyi* and this independently from the glycerol employed (pure or crude). Also the percentage of oleic acid was significantly different: the highest percentage of 18:0 was observed in *L. starkeyi* (~39.4 and ~40.9 % on pure and crude glycerol, respectively), while the lowest percentages were observed in *C. curvatus* (~27.6 and ~31.2 % on pure and crude glycerol, respectively).

GC analysis of *R. toruloides* and *C. curvatus* were performed also just before the end of the fermentations (168 h and 240 h for *R. toruloides* and *C. curvatus*, respectively), finding that fatty acids profiles (% wt/wt) were basically unchanged compared to those observed at 144 h (data not shown).

The lipid chains of all the analyzed yeasts exhibit low degrees of unsaturation, which is desirable for their application in biodiesel production [43]. The highest percentages of polyunsaturated fatty acids (PUFAs) were observed in *R. toruloides* (~20.2 and ~18.0 % on pure and crude glycerol, respectively), while the lower amounts were observed in *L. starkeyi* (~8.1 and ~7.8 % on pure and crude glycerol, respectively).

It is reported in literature that comparing the fatty acids profiles of different oil feedstock [3], the biodiesel derived from yeast lipids are more saturated. It is also reported that yeast oil together with Palm oil and Jatropha oil have the highest percentages of monounsaturated fatty acids, which make them better sources for biodiesel production than soybean oil [21]. Overall, results reported in Table 3 are in line with these observations, showing in all the tested yeasts a higher content of saturated and monounsaturated fatty acids.

We have also observed that, for all the yeasts tested in this study, the percentages of 18:1 were lower than 20 % at day 2 (data not shown), but increased over time reaching values reported in Table 3 by day 7 and were stable thereafter. Over time, the increase in the percentages of oleic acid (18:1) was followed by a decrease of the percentages of stearic acid (18:0): as example, in *R. toruloides* the percentages of 18:0 were ~50.0 % at day 2 but decrease to ~12.0 % by day 7. Percentages of linoleic acid (18:2) changed over time but with some differences among the different yeasts: in *R. toruloides* and *C. curvatus* the percentages decreased from day 2 to day 7 (~10.0 %) while in *L. starkeyi* percentages of 18:2 were very similar over time. Differently, percentages of 14:0, 16:1 and 18:3 were basically unchanged during the entire experimental time.

When considering whether biodiesel derived from yeast lipids is suitable for use as a fuel, several factors should be taken into account. The successful commercialization of biodiesel in many countries has been accompanied by the development of standards, as ASTM D6751 (ASTM = American Society for Testing and Materials) and the European standard EN, to ensure high product quality and user confidence [44]. In particular, ignition quality, heat of combustion, cetane number, oxidative stability, viscosity and lubricity are some of the properties of the biodiesel fuel influenced by fatty acids structure. Over the years predictive equations were developed and have generally proved successful in estimating the physical properties of oil composed of FAMEs [45]. In the present study, the equations reported in [46] were employed to predict biodiesel properties, such as the viscosity and the cetane number. While cetane number for the tested yeasts was in the range between 59–62, meeting the international specifications [45], kinematic viscosity predicted values exceeded the limits of 3.5–5 mm^2^ s^−1^ set by EN 14214.

However, as reported in [1], it is important to remember that the majority of the yeast oil-based biodiesel could be used as fuel for diesel engines only when they are blended with diesel fuel and the fatty acid profile that provides a fuel with all optimal parameters does not exist yet.

## Conclusions

Valorization of waste raw materials represents a scientifically intriguing and socially relevant challenge in industrial biotechnology.

In this work we presented the oleaginous yeasts *R. toruloides*, *C. curvatus* and *L. starkeyi* as three of the most promising cell factories for biodiesel production starting from crude glycerol as substrate. In particular, we have demonstrated the possibility to design an efficient yet simple and robust feeding strategy useful in preventing the inhibitory effect of crude glycerol. The fermentation protocol is not trivial since, as well exemplified by our data on *L. starkeyi*, the transition between growth and lipid accumulation is species-specific.

With the herein described strategy all the selected yeasts were not only able to reach high biomass content but also to efficiently accumulate lipid over time, being high productivity one of the key parameters for the development of a viable process of production. As reported in literature [47] yeast adaptation is one of the possible strategies for dealing with inhibitor problems. In this work, we suggest that the feeding strategy guarantees a progressive cells adaptation to increasing inhibitors concentrations, leading to an improved bioconversion of crude glycerol into lipids. The final lipid production on crude glycerol is similar if not higher than the one reached on pure glycerol. From the comparative analysis *L. starkeyi* showed the highest glycerol consumption rate, while *R. toruloides* the highest productivity and lipid titer.

FTIR microspectroscopy and flow cytometry analyses demonstrated to be efficient and time-saving methods for monitoring lipid accumulation along the process within intact cells. However, GC analysis is still indispensable to accurately identify the lipids extracted from cells. Noteworthy, FTIR microspectroscopy revealed important changes occurring over time in the yeast cell wall composition. Such changes were found to be strain specific, while only negligible spectral differences resulted from the use of pure or crude glycerol as carbon source. This observation is relevant, since it supports that, once avoided the initial inhibition, crude glycerol has not detrimental effects on the yeast growth, including those affecting the chemico-physical properties of the cell wall carbohydrate components. Considering that downstream processing procedures are costly and considering that one of the major issue with oleaginous yeasts is cell breakage to rescue the product, these analyses will be relevant in guiding the choice of the cell factory, in designing the producing protocol, willing to develop a cost-effective process of production.

## Methods

### Strains and media

*R. toruloides* (DSM 4444), *C. curvatus* (DSM 70022) and *L. starkeyi* (DSM70295) were purchased from DSMZ. Yeasts were stored in cryotubes at −80 °C in 20 % glycerol (vv^−1^). The *S. cerevisiae* strain used in this study was GRF18U (MATa; ura3; leu2-3112; his3-11,15; cir^+^, [48]).

The composition of the inoculum and fermentation medium was (per liter): 1 g of yeast extract (0.114 g of nitrogen), 1.31 g of (NH_4_)_2_SO_4_ (0.278 g of nitrogen), 0.95 g of Na_2_HPO_4_, 2.7 g of KH_2_PO_4_, 0.2 g of Mg_2_SO_4_ 7H_2_O, 0.04 g. After the pH was adjusted to 5.5 using NaOH 4 M, the medium was supplemented with a 100X trace mineral stock solution consisting of (per liter): 4 g CaCl_2_ 2H_2_O; 0.55 g FeSO_4_ 7H_2_O; 0.52 g citric acid; 0.10 g ZnSO_4_ 7H_2_O; 0.076 g MnSO_4_ H_2_O; and 100 μL 18 M H_2_SO_4_.

Yeast extract was provided by Biolife Italiana S.r.l., Milan, Italy. All the others reagents were provided by Sigma-Aldrich Co., St Louis, MO, USA.

Glycerol, both crude and pure, was used as carbon and energy source. Accordingly [49], for the calculations of the carbon-to-nitrogen ratio (C/N), a carbon content in glycerol of 39.1 %, and a nitrogen content of 21.2 and 11.4 % in ammonium sulfate and yeast extract, respectively, were assumed.

### Raw material

Crude glycerol derived from industrial biodiesel production out of palm oil. The stock had 80 % content of glycerol and appeared as a dark brown liquid. In the text, reported crude glycerol concentrations (g L^−1^) refer to the HPLC measured glycerol. Composition analyses of this stock were conducted: the original sample was diluted 50-fold and passed through 0.22 μm filter. Glycerol and methanol concentrations were determined by HPLC (see below for specifications). Crude glycerol was centrifuged at 6000 rpm for 20 min. The top, dark red layer, mainly consisting of free fatty acids (FFAs), was removed.

### Batch cultures

Flasks experiments were conducted in 250 mL Erlenmeyer flasks containing 50 mL of liquid medium. As carbon and energy sources, glycerol stocks (pure and crude) were mixed at different ratio to a final concentration of 100 gL^−1^ (for crude glycerol, the calculation was done on the basis of the HPLC measurements). In particular, five different media (from Medium A to Medium E) were tested: 100 % of pure glycerol (Medium A), 80 % of pure and 20 % of crude glycerol (Medium B), 70 % of pure and 30 % of crude glycerol (Medium C), 50 % of pure and 50 % of crude glycerol (Medium D) and 100 % of crude glycerol (Medium E). The pH value of the medium was adjusted to 5.5 with NaOH 4 M and was then maintained using a citrate buffer solution pH5.5 at the final concentration of 0.1 M.

Shake flasks experiments were performed in triplicates on a rotary shaker at 25 °C and 220 rpm. The cultures were initiated upon 45 mL of the cultivation medium inoculated with 5 mL of the seed culture to a final absorbance (OD_660_) of 3.0. Growth was monitored by regularly measuring optical density (OD_660_) over time and by determining the cell dry weight (CDW) at 0 and 216 h (final point). Samples for flow-cytometry and fluorescence microscopy analysis (1 mL) were taken every 24 h. The supernatant of these samples was used for the HPLC analysis.

### Fed-batch cultures

Yeasts were revived from cryo-preserved stocks stored at −80 °C and grown on YP-Glycerol agar plates. One colony was used to inoculate 1 L flasks with 200 mL of the culture medium and seed cultures were placed on a rotary shaker at 25 °C and 220 rpm for 3 days. Exponential phase shake flasks cultures were used to inoculate bioreactors to a final optical density (OD_660_) of 3.0. Briefly, cells were centrifuged at 6000 rpm for 5 min, washed twice with water, and finally resuspended in 20 mL of sterilized water.

The fed-batch experiments were conducted in 2.0 L bioreactors (Sartorius Stedim BIOSTAT^®^ Bplus, Germany). For all bioreactor cultivations, the aeration rate, agitation, and temperature were set to 1 vvm, 300 rpm (in cascade to 25 % of dissolved oxygen), and 25 °C, respectively. The pH was maintained by automatic pumping of 4 M NaOH.

The cultivation was designed to have three different phases: (1) initial phase on pure glycerol (from 0 to 28 h) to adapt cells to the operative conditions; (2) “feeding” phase (from 28 to 48 h) where the medium was fed to reach a final concentration of about 100 gL^−1^ of glycerol (crude versus pure)^;^ (3) “lipid accumulation” phase under nitrogen limitation and in excess glycerol (from 48 h to the time of glycerol depletion).

Bioreactor experiments were started with a working volume of 1.0 L and with 15 gL^−1^ as initial concentration of pure glycerol, resulting in a medium with a balanced C/N ratio.

For each bioreactor the feeding consisted in a 540 mL solution containing concentrated pure or crude glycerol, so that the final glycerol concentration at the end of the feeding was ~100 gL^−1^, and nutrients (salts and traces) to support cell growth. This led to a final working volume of 1.5 L (considering sampling).

The feeding rate was set as fast as possible for reaching the desired C/N ratio but based on the assumption that the biomass concentration should not decrease below the value measured at the beginning of the feeding. The lowest specific growth rate on pure glycerol was taken as reference to determine how long should the feed last. In our case, the (lowest) specific growth rate of 0.0247 h^−1^ was observed with *C. curvatus*. Based on this value the minimal feed length was calculated to be of 20 h corresponding to a feeding rate of 0.45 mL min^−1^. This feeding rate was applied in all fermentations to allow a direct comparison between the yeasts.

When needed, antifoam emulsion (Sigma-Aldrich, MO, USA) was added to prevent excess foam formation. Aliquots were collected at regular intervals to evaluate substrate concentration (HPLC analysis), optical density (OD_660_), cell dry weight (CDW) and lipid content (flow-cytometry, gas chromatography, fluorescence microscopy and FTIR analysis).

### Analytical methods

The optical density was measured at 660 nm (OD_660_) with a Shimadzu UV-1800 spectrophotometer (Shimadzu Corporation). Samples collected at different times were centrifuged at 14000 rpm for 10 min. The supernatants were filtered (0.22 μm filter) and glycerol and methanol concentrations were HPLC determined using a Rezex ROA-Organic Acid (Phenomenex). The eluent was 0.01 M H_2_SO_4_ pumped at 0.5 mL min^−1^ and column temperature was 35 °C. Separated components were detected by a refractive-index detector and peaks were identified by comparing with known standards (Sigma-Aldrich, St Louis, MO, USA).

Biomass was harvested by centrifugation of the culture samples at 4000 rpm for 10 min. The pellets were then washed twice with distilled water and dried at 40 °C (Concentrator 52301, Eppendorf, Germany) until a constant weight was obtained.

Additional biomass was also preserved for cellular lipids and fatty acids analysis.

### Fluorescence microscopy and flow-cytometry analysis

Cell staining for lipids analysis was performed by using Nile Red (9-diethylamino-5-benzo[α]phenoxazinone) obtained from Sigma-Aldrich Co. (St Louis, MO, USA). As Nile Red is sensitive to light [50], it was always handled with reduced light conditions, and stored at 4 °C.

Briefly, a Nile Red stock solution (314 μM) was prepared by dissolving 0.1 mg of Nile Red in 1 mL acetone [30, 51]. Cells were washed twice with PBS buffer (0.05 M, pH 7.0) and Nile Red was then added at a final concentration of 31.4 μM in PBS. Before measurements, cells were incubated for 5 min in the dark at room temperature.

Flow-cytometry analysis were conducted using a Beckman Coulter FC-500 flow cytometer (Beckman Coulter, Fullerton, CA, USA) equipped with an Argon ion laser (excitation wavelength 488 nm, laser power 20 mW). Upon excitation, NR exhibits intense yellow-gold fluorescence when dissolved in neutral lipids, and red fluorescence when dissolved in polar lipids [52]. The optical system used collect red light (>650 nm corresponding to polar lipids) in the FL3 channel. A total of 20.000 cells were measured for each sample using a log amplification of the fluorescent signal. Non-stained cells were used as auto-fluorescence control. Data analysis was performed afterwards with *Flowing software* (http://www.flowingsoftware.com) and *Cyflogic* (http://www.cyflogic.com).

Fluorescence microscopy studies were carried out with a Nikon Eclypse 90i (Nikon Instruments, Inc.). Nile Red fluorescence was registered using a 515- to 560-nm band-pass exciter filter. Images of stained cells were acquired both in dichroic and fluorescence mode.

### Gas chromatography analysis

To determine the lipid content in yeast cells, lipids were extracted, based on the method of Bligh and Dyer [53] with modifications, and then analyzed through GC. Briefly, 10 OD (about 5 × 10^8^ cells) of samples were centrifuged at 4000 rpm for 10 min and washed twice with 1 mL of distilled water. Pellets were then resuspended in 5 mL of MeOH/CHCl_3_ (2:1) and mechanically disrupted twice using a French Press at 38.000 psi (Constant Cell Disruption System, Constant System Ltd). Then, 2 mL of citric acid and 3 mL of CHCl_3_ were added to the samples. After mixing, the samples were centrifuged at 4000 rpm for 2 min and the upper phase was discarded. Derivation of methyl esters from fatty acids was as previously described [54]. Fatty acid methyl esters were analysed by gas chromatography (DANI GC 1000. Alltech ECONO-CAPTM ECTM-WAX column, 30 m × 0.32 mm ID × 0.25 μm). Starting from 100 °C, the column temperature was headed to 200 at 10 °C min^−1^, temperature was further increased to 245 at 5 °C min^−1^ and then maintained for 1 min. Nonanoic acid was used as an internal standard. Fatty acids were identified by comparison of their retention times with those of standard (Sigma-Aldrich, St Louis, MO, USA), quantified based on their respective peak areas and normalized.

### FTIR microspectroscopy

Yeast cells from *S. cerevisiae*, *C. curvatus*, *L. starkeyi* and *R. toruloides*, at different time points (T = 0, T = 28, T = 48, T = 72, T = 144, T = 168 and T = 240 h from the inoculum) were washed three times in distilled water to eliminate medium contamination. Approximately 3 μL of the cell suspensions were then deposited onto an IR transparent BaF_2_ support, and dried at room temperature for at least 30 min to eliminate the excess water. FTIR absorption spectra were acquired in transmission mode, between 4.000 and 700 cm^−1^, by means of a Varian 610-IR infrared microscope coupled to the Varian 670-IR FTIR spectrometer (both from Varian Australia Pty Ltd), equipped with a mercury cadmium telluride (MCT) nitrogen-cooled detector. The variable microscope aperture was adjusted from approximately 60 × 60 to 100 × 100 μm. Measurements were performed at 2 cm^−1^ spectral resolution; 25 kHz scan speed, triangular apodization, and by the accumulation of 512 scan co-additions. When necessary, spectra were corrected for residual water vapour absorption [32, 55].

Spectral analysis was conducted in the spectral range between 4.000 and 800 cm^−1^. To this aim, second derivative spectra were obtained following the Savitsky-Golay method (third-grade polynomial, nine smoothing points), after a binomial 13 smoothing points of the measured spectra, using the GRAMS/32 software (Galactic Industries Corporation, USA).

To verify the reproducibility and reliability of the spectral results, more than three independent preparations were analyzed. In the Figures, reported data are representative of the independent experiments performed.

### Principal component analysis of FTIR data

The PCA was independently performed on three ranges, 3050–2800, 1800–1350, and 1350–900. Since each range provides different and specific information, splitting the analysis on different ranges allows an easier interpretation of the PCA results. The correlation matrix was computed on standardized spectra (zero mean and standard deviation equal to 1) and diagonalized to get eigenvectors (loadings, *v*) sorted according to the magnitude of the corresponding eigenvalues [56]. In all cases, the first three eigenvectors already describe more than 90 % of the total variance of the data. Principal components (scores) have been obtained projecting the original spectra on the orthogonal subspace defined by the first three eigenvalues. To quantify the distance from the lipids standards, the Euclidean distance in the three-dimensional principal components space was used:

$$d\left( {\varvec{p}_{i}^{{K_{t} }} ,\varvec{p}_{j}^{S} } \right) = \mathop \sum \limits_{m = 1}^{3} \sqrt {\left( {p_{m,i}^{{K_{t} }} - p_{m,j}^{S} } \right)^{2} }$$

where **p** indicates 3D principal component, *K* refers to the strains (*R. toruloides, C. curvatus, S. cerevisiae, L. starkeyi*), t corresponds to the time of growth (0 h or 144 h), m is the m-th principal component and *S* indexes the standard lipids. Since each sample was measured multiple times the distance is computed between the *i*-th replica of sample *K* and the *j*-th replica of the standard S, obtaining the matrix $\varvec{D}^{{K_{t} ,S}} = \left[ {d\left( {\varvec{p}_{i}^{{K_{t} }} ,\varvec{p}_{j}^{S} } \right)} \right]$. An average value for the distance between *K* and *S* was obtained computing the median of all the *N(N*-*1)/2 i*-*j* pairs, $\tilde{d}\left( {K_{t} ,S} \right) = median\left( {\varvec{D}^{{K_{t} ,S}} } \right).$ Moreover to better quantify how much samples differ in terms of distance with the standard lipids, the percentage change between time 144 and 0 has been computed as:

$$\Updelta \left( {K_{144h;0h} ,S} \right) = \left[ {1 - \frac{{\tilde{d}\left( {K_{144h} ,S} \right)}}{{\tilde{d}\left( {K_{0h} ,S} \right)}}} \right] \times 100$$

A positive value indicates that a given lipid standard contributes to the spectral profile changes of the sample at 144 h compared to the time 0; this suggests that it is accumulated during growth. Since all the procedure is repeated for the three analyzed ranges, we indicate the percentage change as Δ(*K*_144*h*;0*h*_, *S*)_*R*_, where R makes clear the range dependence. Finally, a unique average percentage change for each *K*-*S* pair is obtained averaging over the ranges. $\tilde{\Updelta }\left( {K_{{144h;0h}} ,S} \right) = \frac{1}{R}\sum\nolimits_{R} {\Updelta \left( {K_{{144h;0h}} ,S} \right)_{R} }$.

Uncertainties have been estimated using a bootstrap procedure [57]. In particular, using 1000 bootstrap iterations, confidence intervals on the range specific percentage change, $\Updelta \left( {K_{144h;0h} ,S} \right)_{R} ,$ have been obtained using the bootstrap percentile method [58]. All data analyses were performed using the software package R [59], version 3.0.2. A pseudocode of the algorithm used for the bootstrap-PCA [57] we have implemented is given in the Additional file 1 section (from Figures S4–S6).

## Additional file

10.1186/s12934-016-0467-x Additional Figures and Tables; Figures S1–S6, Tables S1–S2. Additional information; optimization of the feeding strategy.

## Abbreviations

C/N: carbon-to-nitrogen
CDW: cell dry weight
NR: nile red
FA: fatty acids
FAME: fatty acids methyl esters
FTIR: fourier transform infrared
GC: gas chromatography
IR: infrared
OD: optical density
PBS: phosphate-buffered saline
PCA: principal component analysis
