# Supplementary material for: Assessing an effective feeding strategy to optimize crude glycerol utilization as sustainable carbon source for lipid accumulation in oleaginous yeasts
Source: Microb Cell Fact. 2016 May 5;15:75. doi: 10.1186/s12934-016-0467-x (PMC4858929; doi:10.1186/s12934-016-0467-x)
Supplement: Supplementary file 1 — 10.1186/s12934-016-0467-x Additional Figures and Tables; Figures S1–S6, Tables S1–S2. Additional information; optimization of the feeding strategy. [file 12934_2016_467_MOESM1_ESM.docx]

**Additional files for:**

*Microbial Cell Factories*

**Assessing an effective feeding strategy to optimize crude glycerol utilization as sustainable carbon source for lipid accumulation in oleaginous yeasts**

Lorenzo Signori^1^, Diletta Ami^1,2,3^, Riccardo Posteri^1^, Andrea Giuzzi^1^, Paolo Mereghetti^4^, Danilo Porro^1^, Paola Branduardi^1§^

^1^ Department of Biotechnology and Biosciences, University of Milano-Bicocca, Piazza della Scienza 2, Milano 20126 (Italy).

^2^ Department of Physics, University of Milano-Bicocca, Piazza della Scienza 3, Milano 20126 (Italy).

^3^ Consorzio Nazionale Interuniversitario per le Scienze fisiche della Materia (CNISM) UdR Milano-Bicocca, Via R. Cozzi 53, Milano 20126 (Italy).

^4^ Center for Nanotechnology Innovation@NEST, Istituto Italiano di Tecnologia, Piazza San Silvestro 12, Pisa 56127 (Italy).

^§^Corresponding author

Email addresses:

LS: l.signori@campus.unimib.it

DA: diletta.ami@unimib.it

RP: riccardo.posteri@unimib.it

AG: a.giuzzi@campus.unimib.it

PM: paolo.mereghetti@gmail.com

DP: danilo.porro@unimib.it

PB: paola.branduardi@unimib.it

**Assessing the feeding strategy for minimizing the inhibitory effect of crude glycerol**

As described in the manuscript, different feeding rates were tested. Herein, as representative example of the feeding optimization phase, three feeding rates applied to the yeast *L. starkeyi* are reported*.*

**
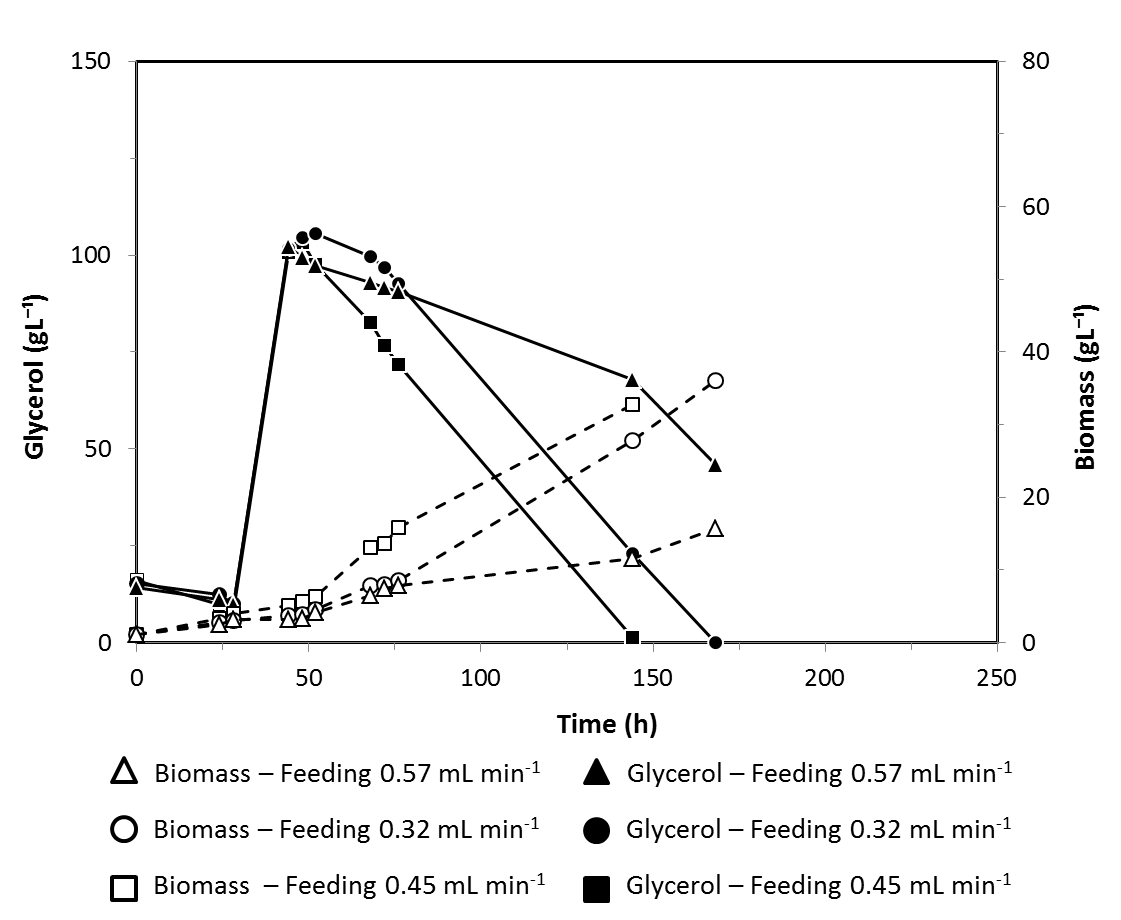
**

**Figure S1 – Crude glycerol consumption and growth (CDW) profiles of *L. starkeyi* under different feeding rate.**

Growth (CDW; g L^-1^; dashed lines) and crude glycerol consumption profiles (g L^-1^; continuous line) of *L. starkeyi* under three different feeding rate: 0.57 mL min^-1^ (Triangles), 0.45 mL min^-1^ (Squares) and 0.32 mL min^-1^ (Circles).

As shown in Figure S1, with all the tested feeding rates *L. starkeyi* was able to grow on crude glycerol avoiding the growth inhibition observed during batch cultivations. The slower crude glycerol consumption was observed with the highest feeding rate (0.55 mL min^-1^) among the tested ones: after 168 h only 55 g L^-1^ of crude glycerol were consumed. Instead, the faster crude glycerol consumption was observed with the feeding rate of 0.45 mL min^-1^.

The biomass production rate was directly related to the glycerol consumption rate, being maximal with the feeding rate of 0.45 mL min^-1^. However, the highest biomass production was reached with the slower feeding rate (0.32 mL min^-1^).

Overall, among the tested ones, the feeding rate of 0.45 mL min^-1^ was found to be the most effective in terms of lipid productivity and lipid content (on dry cell weight basis).


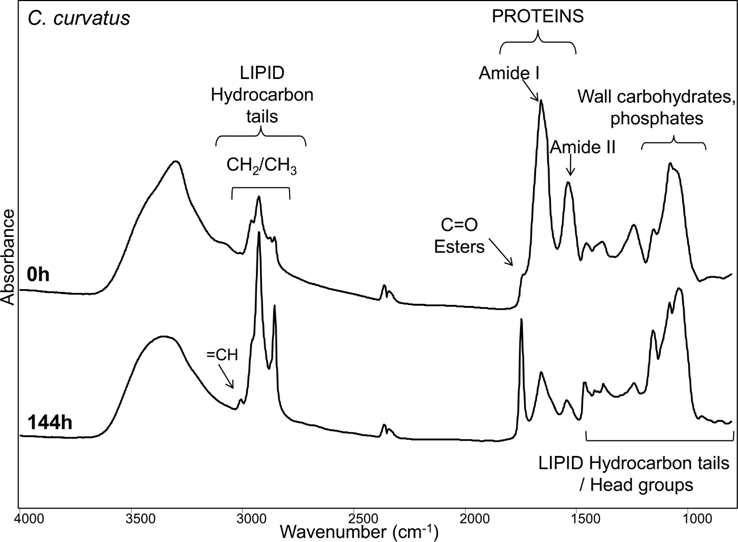


**Figure S2 – FTIR spectra of *C. curvatus* intact cells.**

The measured IR absorption spectra of *C. curvatus* cells at 0 and 144 hours of growth are displayed. The assignment of selected bands to the main biomolecules is reported.


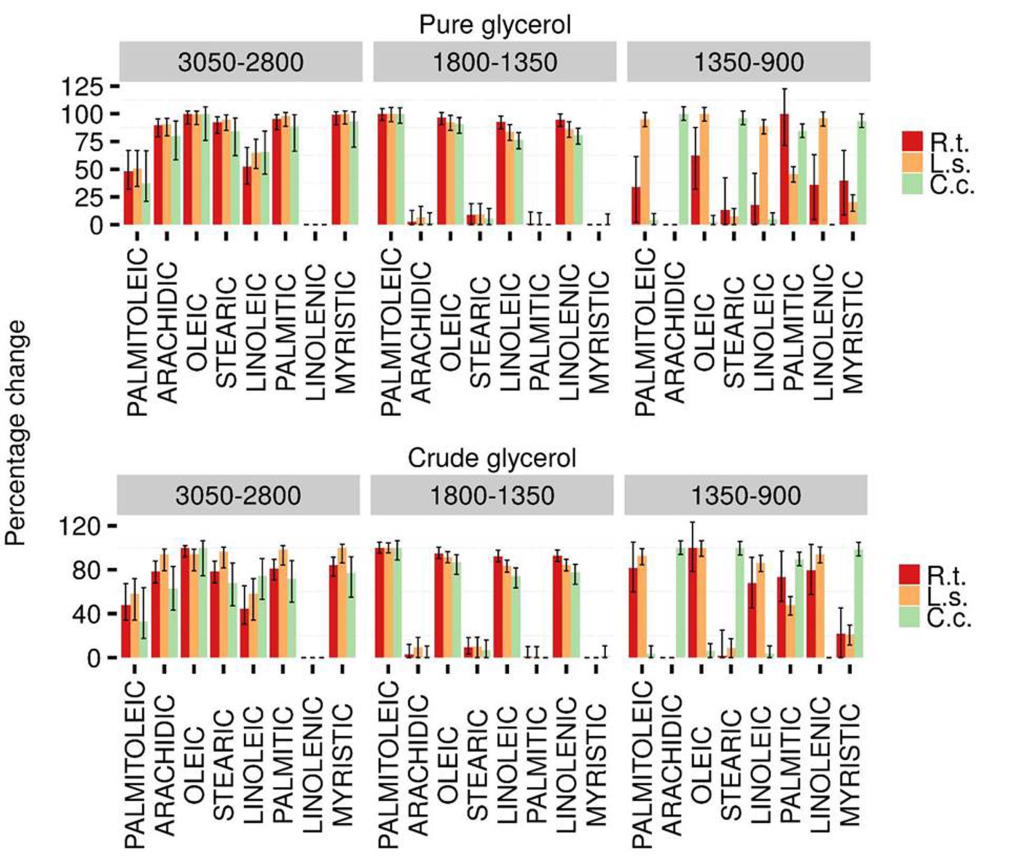


**Figure S3 – Percentage changes.**

Percentage change (${\Delta\left( K_{144h;0h},S \right)}_{R}$, see Methods) are shown for each analyzed range. A high value suggests that a given lipid is accumulated during growth. Error bars indicate the bootstrapped 95% confidence intervals.

Data are shown for *R. toruloides* (R.t.), *L. starkeyi* (L.s.), and *C. curvatus* (C.c.) grown in medium supplemented with pure glycerol (top panels) and crude glycerol (bottom panels).


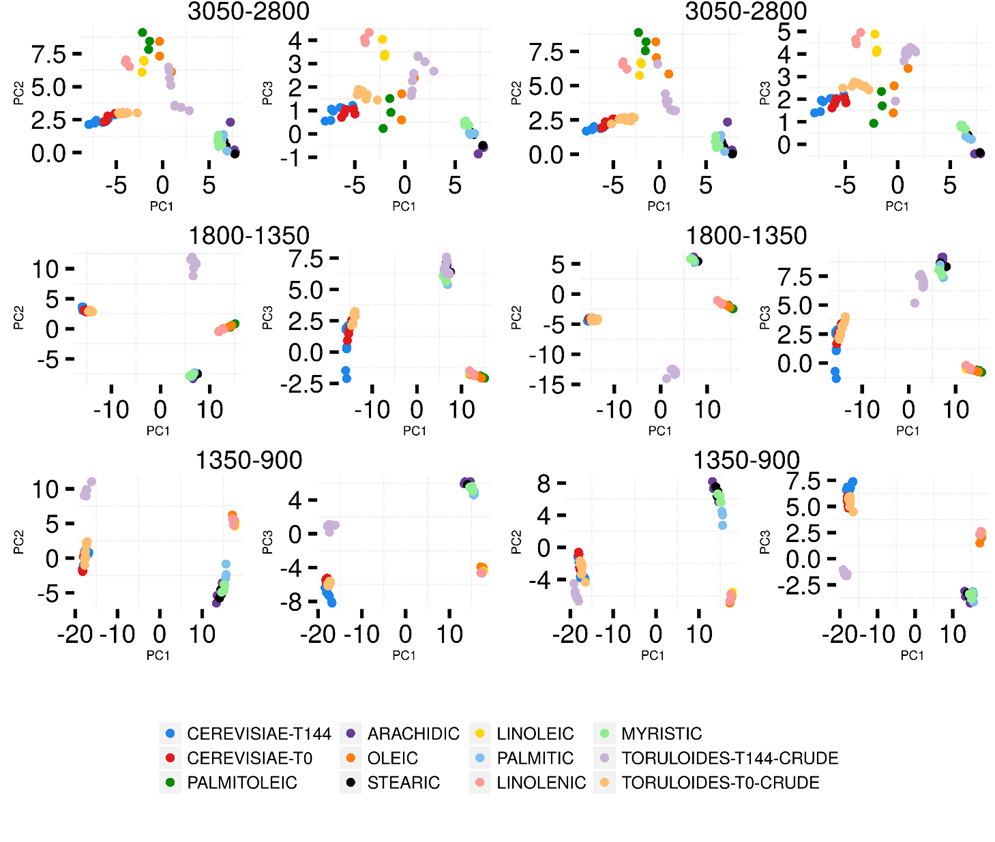


**Figure S4 – PCA score plots, *R. toruloides.***

Bootstrapped PCA two-dimensional score plots of the FTIR absorption spectra of *R. toruloides* and *S. cerevisiae* intact cells, and of the selected fatty-acid standards, performed in three different ranges. Growth medium supplemented with crude glycerol (left panels), growth medium supplemented with pure glycerol (right panels).


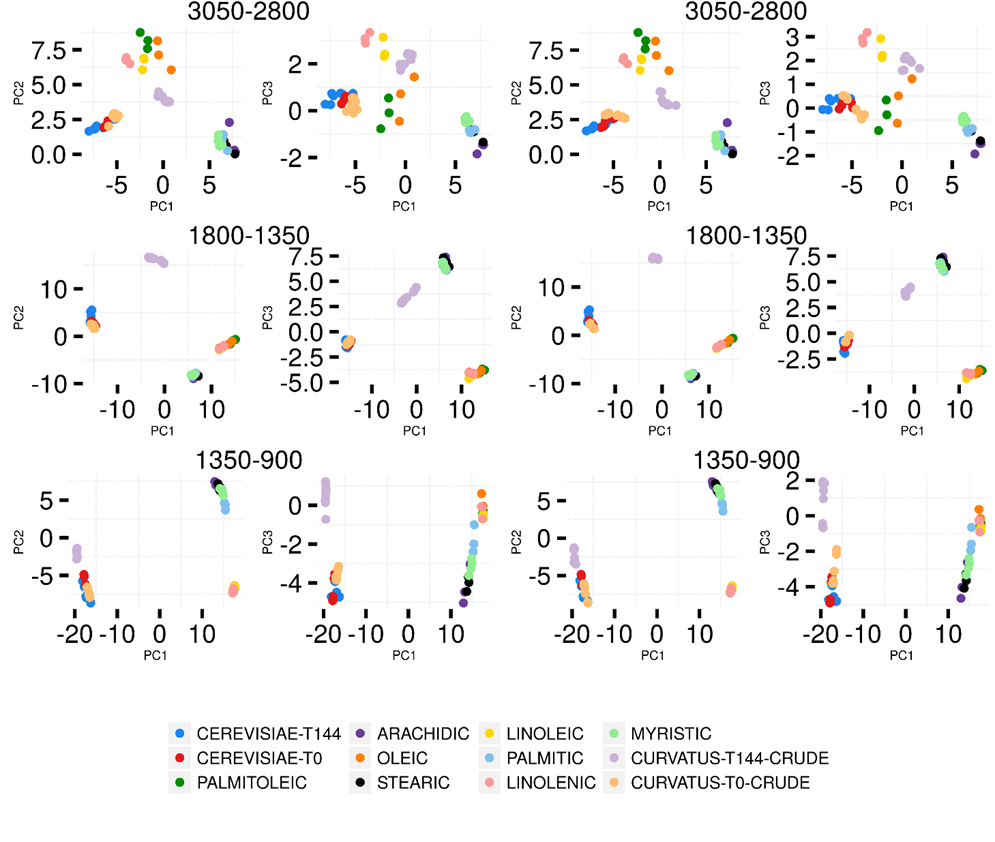


**Figure S5 – PCA score plots, *C. curvatus*.**

Bootstrapped PCA two-dimensional score plots of the FTIR absorption spectra of *C. curvatus* and *S. cerevisiae* intact cells, and of the selected fatty-acid standards, performed in three different ranges. Growth medium supplemented with crude glycerol (left panels), growth medium supplemented with pure glycerol (right panels).


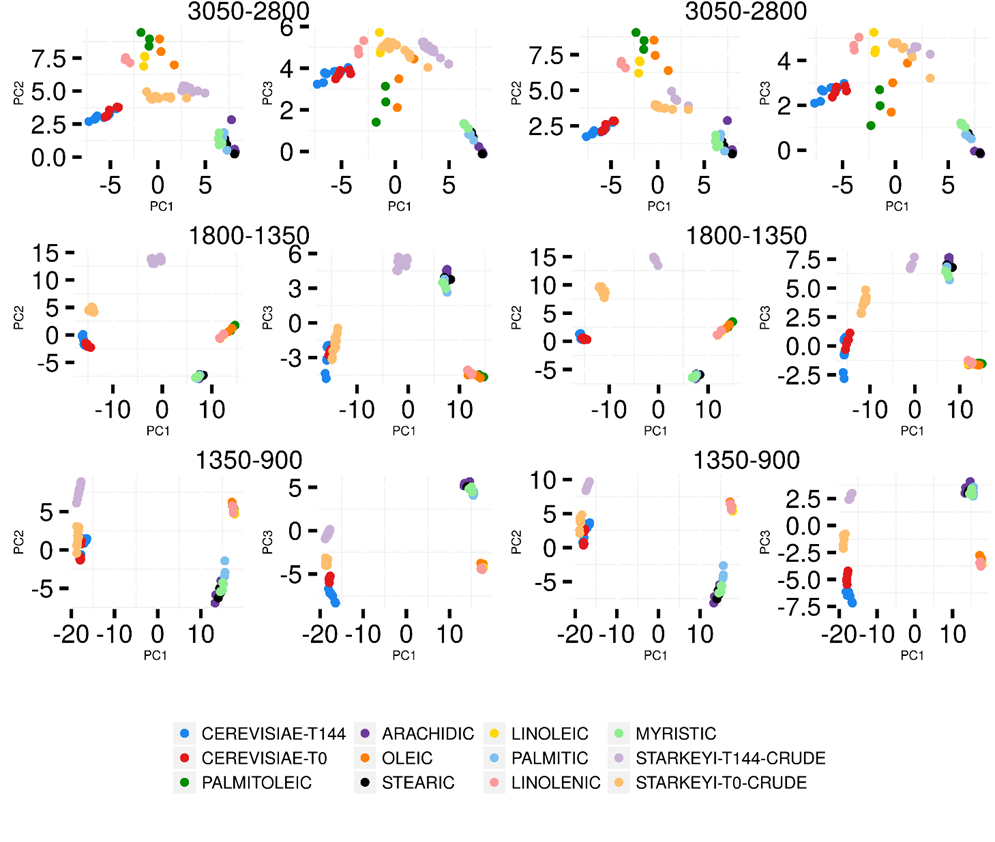


**Figure S6 – PCA score plots, *L. starkeyi*.**

Bootstrapped PCA two-dimensional score plots of the FTIR absorption spectra of *L. starkeyi* and *S. cerevisiae* intact cells, and of the selected fatty-acid standards, performed in three different ranges. Growth medium supplemented with crude glycerol (left panels), growth medium supplemented with pure glycerol (right panels).

**Table S1 – Comparison of optical density (OD_660_) and cell dry weight (CDW) values of *R. toruloides*, *C. curvatus* and *L. starkeyi* during shake flasks experiments performed in different media (from Medium A to Medium E).**

| **Yeast** | **Medium** |  | **OD (660 nm)** | **CDW (g L ^-1^)** |  | **OD (660 nm)** | **CDW (g L ^-1^)** |
| --- | --- | --- | --- | --- | --- | --- | --- |
|  |  |  | **0 h** | |  | **216 h** | |
|  |  |  |  |  |  |  |  |
| ***R. toruloides*** | **A** |  | 3.3 | 1.1 |  | 70.0 | 26.5 |
|  | **B** |  | 3.0 | 1.0 |  | 88.8 | 30.3 |
|  | **C** |  | 3.3 | 1.1 |  | 84.0 | 28.6 |
|  | **D** |  | 3.4 | 1.1 |  | 3.7 | 1.3 |
|  | **E** |  | 3.4 | 1.1 |  | 0.8 | 0.3 |
|  |  |  |  |  |  |  |  |
| ***C. curvatus*** | **A** |  | 2.9 | 0.9 |  | 63.9 | 19.8 |
|  | **B** |  | 3.3 | 1.0 |  | 64.0 | 19.9 |
|  | **C** |  | 2.9 | 0.9 |  | 9.6 | 3.0 |
|  | **D** |  | 2.9 | 0.9 |  | 3.1 | 1.0 |
|  | **E** |  | 2.9 | 0.9 |  | 3.0 | 0.9 |
|  |  |  |  |  |  |  |  |
| ***L starkeyi*** | **A** |  | 3.0 | 1.2 |  | 59.0 | 22.4 |
|  | **B** |  | 2.8 | 1.2 |  | 65.8 | 25.2 |
|  | **C** |  | 2.8 | 1.2 |  | 5.9 | 2.2 |
|  | **D** |  | 2.8 | 1.2 |  | 3.4 | 1.3 |
|  | **E** |  | 2.8 | 1.2 |  | 3.0 | 1.2 |

Data shown are the mean of three independent experiments where the deviation from the mean value was for most values less than 5 %.

**Table S2 – X mean values, representing the medium forward scatter (FS) and side scatter amplification (SS), of *R. toruloides*, *C curvatus* and *L. starkeyi* during shake flasks experiments performed in MediumA.**

| **Yeast** |  | **SS (X mean)** | | | |  | **FS (X mean)** | | | |
| --- | --- | --- | --- | --- | --- | --- | --- | --- | --- | --- |
|  |  | **24 h** | **72 h** | **144 h** | **192 h** |  | **24 h** | **72 h** | **144 h** | **192 h** |
|  |  |  |  |  |  |  |  |  |  |  |
| ***R. toruloides*** |  | 32.8 | 50.7 | 64.4 | 60.3 |  | 71.9 | 111.4 | 121.7 | 121.8 |
|  |  |  |  |  |  |  |  |  |  |  |
| ***C. curvatus*** |  | 30.8 | 46.5 | 67.9 | 76.8 |  | 51.4 | 53.7 | 76.8 | 100.0 |
|  |  |  |  |  |  |  |  |  |  |  |
| ***L. starkeyi*** |  | 81.5 | 88.5 | 90.5 | 141.5 |  | 149.2 | 163.9 | 161.7 | 182.7 |

FS: forward scatter

SS: side scatter

Results shown are representative of three independent experiments, where the deviation from the X mean value was always less than 5 %.

Flow-cytometry analysis showed that cells expanded in size over time, concurrently with the lipid accumulation confirmed by Nile Red staining: as example, during shake flasks experiments performed in Medium A in the three yeasts was observed an increase of the forward scatter (FS) signal over time (Table S2). Moreover, in Table S2 it is also possible to observe a progressive increase of the side scatter (SS) signal, probably due to the lipid bodies formation.
